# A novel *Meloidogyne graminicola* effector, MgGPP, is secreted into host cells and undergoes glycosylation in concert with proteolysis to suppress plant defenses and promote parasitism

**DOI:** 10.1371/journal.ppat.1006301

**Published:** 2017-04-12

**Authors:** Jiansong Chen, Borong Lin, Qiuling Huang, Lili Hu, Kan Zhuo, Jinling Liao

**Affiliations:** 1 Laboratory of Plant Nematology, South China Agricultural University, Guangzhou, China; 2 Guangdong Province Key Laboratory of Microbial Signals and Disease Control, South China Agricultural University, Guangzhou, China; 3 Guangdong Eco-Engineering Polytechnic, Guangzhou, China; Stanford University, UNITED STATES

## Abstract

Plant pathogen effectors can recruit the host post-translational machinery to mediate their post-translational modification (PTM) and regulate their activity to facilitate parasitism, but few studies have focused on this phenomenon in the field of plant-parasitic nematodes. In this study, we show that the plant-parasitic nematode *Meloidogyne graminicola* has evolved a novel effector, MgGPP, that is exclusively expressed within the nematode subventral esophageal gland cells and up-regulated in the early parasitic stage of *M*. *graminicola*. The effector MgGPP plays a role in nematode parasitism. Transgenic rice lines expressing MgGPP become significantly more susceptible to *M*. *graminicola* infection than wild-type control plants, and conversely, *in planta*, the silencing of MgGPP through RNAi technology substantially increases the resistance of rice to *M*. *graminicola*. Significantly, we show that MgGPP is secreted into host plants and targeted to the ER, where the *N*-glycosylation and C-terminal proteolysis of MgGPP occur. C-terminal proteolysis promotes MgGPP to leave the ER, after which it is transported to the nucleus. In addition, *N*-glycosylation of MgGPP is required for suppressing the host response. The research data provide an intriguing example of *in planta* glycosylation in concert with proteolysis of a pathogen effector, which depict a novel mechanism by which parasitic nematodes could subjugate plant immunity and promote parasitism and may present a promising target for developing new strategies against nematode infections.

## Author summary

Post-translational modification (PTM) is a tool used by prokaryotic and eukaryotic cells to regulate protein activity, and many unique and important functions of proteins depend on appropriate PTMs. Evidence is emerging that plant pathogen effectors can utilize the host post-translational machinery to mediate their PTM and regulate their activity to facilitate parasitism. However, these biochemical modifications have been described only for a limited number of plant-parasitic nematode effectors. In this report, we identified the novel *Meloidogyne graminicola* effector MgGPP, which is important for nematode parasitism. We found that the effector MgGPP is secreted into host tissues and is subjected to glycosylation in concert with proteolysis in rice. Furthermore, we have shown that the proteolytical processing of MgGPP could change the subcellular trafficking of MgGPP, and the *N*-glycosylation of MgGPP can activate its function to suppress resistance gene (RBP-1/Gpa2)-mediated cell death, revealing a strategy of host-mediated PTM that is cleverly exploited by plant-parasitic nematodes to subjugate plant immunity and thereby promote parasitism.

## Introduction

Root-knot nematodes (RKNs) are one of the most economically important plant-parasitic nematodes (PPNs), infecting more than 5500 plant species [1,2]. The soil-borne RKNs devastate varieties of crop plants, resulting in about $70 billion losses in worldwide agriculture annually [3]. Generally, the second-stage juveniles (J2s) of RKNs penetrate host roots and migrate intercellularly towards the vascular cylinder, where they transform five to seven cells around their head into large and multinucleated feeding cells called giant cells that provide RKNs with nutrients and are essential for their development and reproduction [4]. RKNs have evolved numerous effectors that originate from the nematode esophageal gland cells and are secreted into host plant tissues, playing key roles in root invasion and the formation and maintenance of giant cells, resulting in the successful parasitism of RKNs [5].

Decades of research have demonstrated the roles of some effectors of PPNs. For example, extracellular effectors, such as ß-1,4-endoglucanase and pectate lyase, can degrade and depolymerize the main structural polysaccharide constituents of the plant cell wall [6,7], and cyst nematode-secreted CLAVATA3/ESR CLE-like proteins (CLEs) mimic endogenous host-plant CLE peptides [8]. Recently, one of the most exciting discoveries has been that effectors of PPNs are capable of suppressing host defenses directly [9–12]. Previous studies showed that plants have evolved a set of immune system defenses against plant pathogens [13]. When the immune system detects pathogens, a series of immune responses, such as Ca^2+^ spikes, callose deposition, reactive oxygen species bursts, a localized hypersensitive response (HR) and the induction of pathogenesis-related gene expression, can be activated [14,15]. PPNs therefore have also evolved a class of effectors to suppress the host immune system for survival. The first nematode-secreted effector that was found to have the ability to suppress the plant defense responses is the calreticulin Mi-CRT identified in *M*. *incognita* [11]. Subsequently, several effectors, mainly cyst nematode-secreted and root-knot nematode-secreted effectors, such as SPRYSEC-19 and GrUBCEP12 in *Globodera rostochiensis*, Ha-ANNEXIN in *Heterodera avenae*, MeTCTP in *M*. *enterolobii* and MiMsp40 in *M*. *incognita*, were demonstrated to suppress host defense responses directly [9,12,16]. Of these effectors, GrUBCEP12 was found to be cleaved *in planta* [12], suggesting that nematode-secreted effectors may be subjected to post-translational modification (PTM) *in planta*.

PTM, including phosphorylation, acetylation, glycosylation, proteolysis and ubiquitination, is a tool used by prokaryotic and eukaryotic cells to regulate protein activity or promote protein/protein interactions [17,18]. Of the various types of PTM, glycosylation and proteolysis are the two important modes of protein modification in plant cells. *N*-glycosylation has been widely identified and found to play vital roles in diverse aspects of development and physiology, such as the regulation of protein folding, salt tolerance, cellulose biosynthesis, environmental stress responses and plant immunity [19,20]. Intriguingly, the *N*-glycosylation of plant pathogen effectors also plays a role in the infection and parasitism of pathogens [19]. Pathogens can secrete glycoproteins directly into host plants or utilize the host post-translational machinery to form glycosylated effectors, avoiding the plant immunity and promoting pathogenesis of pathogens. For example, a secreted LysM protein, Slp1, was shown to function in *Magnaporthe oryzae* as an effector protein that suppresses host immunity by binding chitin oligosaccharides; however, incomplete *N*-glycosylation of Slp1 led to a dramatic reduction in its chitin-binding capability [19].

Proteolysis is a selective mechanism that can either be co-translational or act in concert with other PTMs in many cellular processes, such as the stress response, maturation of inactive hormones, neuropeptides and growth factors, and targeting of intracellular proteins [21,22]. Post-translational proteolytic processing of plant pathogen effectors *in planta* has been reported. For example, the effector AvrRpt2 from *Pseudomonas syringae* was delivered into host cells via the type III secretion system, where it was specifically cleaved to generate a functional C-terminal end [23].

Previous studies on fungal and bacterial effectors have partly contributed to the understanding of PTM of plant pathogen effectors *in planta*. However, only three nematode-secreted effectors have been found to be post-translationally modified *in planta* as yet. In addition to GrUBCEP12 mentioned above, a CLE effector from *G*. *rostochiensis* and the effector protein 10A07 from *Heterodera schachtii* were glycosylated and phosphorylated *in planta*, respectively [24,25]. It is essential to explore more nematode-secreted effectors with PTM capabilities *in planta* to understand their roles during nematode parasitism.

Rice is the staple food of more than half of the world’s population, and it is also an excellent model system for studying physiological and molecular interactions between plants and PPNs [26,27]. *Meloidogyne graminicola*, one of the most important RKNs, is considered to be a major threat to rice and has caused substantial destruction to up to 87% of the production [28]. Transcriptomes of the rice root-knot nematode *M*. *graminicola* have been obtained [29,30], greatly facilitating the exploration of candidate effectors. However, little is known about *M*. *graminicola* effectors.

Here, we report the cloning and characterization of a novel gene from *M*. *graminicola*. We present several lines of evidence to show that this novel gene affects *M*. *graminicola* parasitism. Additionally, we also provide evidence that the effector encoded by the novel gene can be secreted into host cells, transported from the endoplasmic reticulum (ER) to the nucleus, and post-translationally glycosylated and proteolytically cleaved in host cells. Moreover, only the glycosylated effector is capable of suppressing the host defense response. The effector protein was named MgGPP because of its glycosylation in concert with proteolysis *in planta*.

## Results

### Cloning and sequence analysis of the *M*. *graminicola MgGPP* gene

A 759-bp genomic fragment, designated MgGPP, was obtained. The *MgGPP* gene includes an open reading frame (ORF) of 675 bp (GenBank accession number KY113086), separated by two introns of 43 bp and 41 bp (S1A Fig). The intron/exon boundaries have a conserved 5’-GT-AG-3’ intron splice-site junction [31]. The ORF encodes a 224-amino-acid polypeptide with a predicted molecular size of 25.5 kDa. The protein contains a secretion signal peptide of 20 amino acids at its N-terminus according to the SignalP program and has no putative transmembrane domain based on TMHMM, suggesting that MgGPP may be a secreted protein. MgGPP is predicted to have one *N*-glycosylation site at Asn-110 (Asn-Asp-Ser-Asp, NDSD) and contain a putative SV40-like nuclear localization signal (NLS) domain (^21^EIKKYKP^27^) (S1B Fig), and based on PSORTII, MgGPP is predicted to have a 73.9% probability of being located in the nucleus. Southern blot analysis showed that *MgGPP* is a single-copy gene in the genome of *M*. *graminicola* (S2 Fig).

A BLAST search did not reveal any significant *MgGPP* homologues at the nucleotide level in other organisms but showed matches with several *Meloidogyne* avirulence protein family (MAPs) at the peptide level. However, the shared identities between MgGPP and the MAPs were only 37.3%-41.1%. Moreover, the conserved double-psi beta-barrel domain and repetitive motifs of 13 and 58 aa that exist in the MAPs were not found in MgGPP according to InterProScan. These observations suggest that *MgGPP* is a novel gene of *M*. *graminicola*.

### MgGPP is expressed in the subventral esophageal glands and up-regulated in the early parasitic stage of *M*. *graminicola*

**Fig 1:**
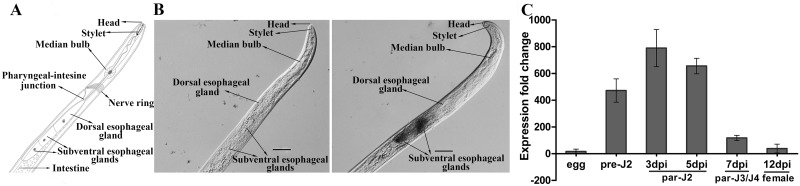
Expression patterns of *MgGPP* in *Meloidogyne graminicola*. (A) Schematic representation of *M*. *graminicola* pre-J2. (B) Localization of *MgGPP* in the subventral esophageal glands of *M*. *graminicola* pre-J2s by *in situ* hybridization. Fixed nematodes were hybridized with (left) sense and (right) antisense cDNA probes from *MgGPP*. Scale bars, 10 μm. (C) The developmental expression pattern of MgGPP by RT-qPCR analysis in five different life stages of *M*. *graminicola*. The fold change values were calculated using the 2^-ΔΔCT^ method and presented as the change in mRNA level at various nematode developmental stages relative to that of the egg stage. The data shown are the means of three repeats plus standard deviation (SD), and three independent experiments were performed with similar results. dpi, days post-infection; pre-J2, pre-parasitic second-stage juvenile; par-J2, par-J3 and par-J4, parasitic second-, third- and fourth-stage juveniles, respectively.

The tissue localization of *MgGPP* in *M*. *graminicola* was investigated using *in situ* hybridization. Strong signals from accumulated transcripts were observed in the subventral esophageal gland cells of *M*. *graminicola* preparasitic second-stage juveniles (pre-J2s) after hybridization with the digoxigenin-labeled antisense ssDNA probe. No signal was detected in pre-J2s when using the sense ssDNA probe as a negative control (Fig 1A and 1B).

The transcriptional expression of the *MgGPP* gene in different stages was analyzed using quantitative real-time PCR (qRT-PCR). The expression level of *MgGPP* at the egg stage was set at one as a reference for calculating the relative fold changes in the other stages. The transcription levels in pre-J2s and parasitic second-stage juveniles (par-J2s) at 3 and 5 days post-infection (dpi) were relatively high. The transcript expression reached a maximum at 3 dpi, with a 789-fold increase in expression compared with the egg stage. The relative fold changes for *MgGPP* transcripts in par-J2s at 5 dpi and in pre-J2s were approximately 655 and 470, respectively, compared with that in the egg stage. After the par-J2 stage, the transcript level of *MgGPP* was dramatically reduced and reached a minimum at the female stages, where only a 38-fold higher transcript level was found compared with the transcripts in the egg stage (Fig 1C). These findings suggested that *MgGPP* may be a secretory protein and play a role in the early stages of *M*. *graminicola* parasitism.

### MgGPP is secreted into plants and targeted to the nuclei of giant cells during parasitism

To determine whether MgGPP is actually secreted within host plants, immunolocalization was performed on the gall sections from rice plants at 5 dpi with *M*. *graminicola* using an antiserum against MgGPP. Western blot analysis was used to determine the serum specificity to MgGPP, which showed a clear hybridizing band with the expected size of ~25.5 kDa in the total protein samples from pre-J2s but not in the protein sample from healthy rice roots. By contrast, the control western blot hybridized with pre-immune serum did not generate any visible band from the nematode and rice root total protein samples (S3 Fig). The results showed that the anti-MgGPP serum can specifically recognize MgGPP of *M*. *graminicola*.

**Fig 2:**
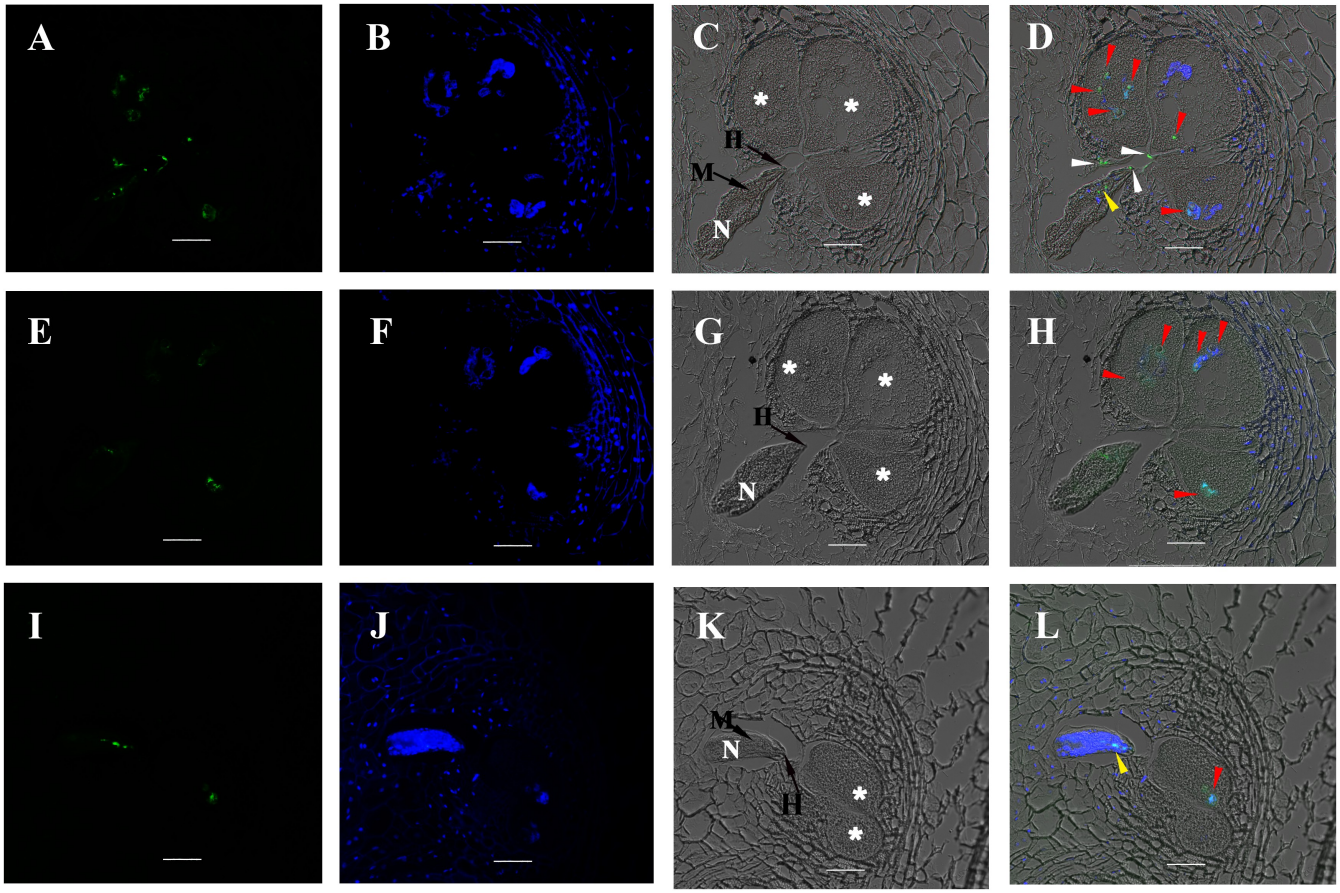
MgGPP localization in sectioned rice root galls at 5 dpi. (A-D) Localization of the secreted MgGPP protein in the giant cell nuclei (red arrows), the cell wall of adjacent giant cells (white arrows) and the lumen of the anterior esophagus of the nematode (yellow arrows). (E-H) Localization of the secreted MgGPP protein in the giant cell nuclei (red arrows). (I-L) Localization of the secreted MgGPP protein in the giant cell nucleus (red arrow) and the lumen of the anterior esophagus of the nematode (yellow arrows). Micrographs A, E and I are observations of the Alexa Fluor 488-conjugated secondary antibody. Micrographs B, F and J are images of 4,6-diamidino-2-phenylindole (DAPI)-stained nuclei. Micrographs C, G and K are images of differential interference contrast. Micrographs D, H and L are superpositions of images of the Alexa Fluor 488-conjugated secondary antibody, DAPI-stained nuclei and differential interference contrast. N, nematode; asterisks, giant cells; M, metacorpus; H, the head of *M*. *graminicola*; Scale bars, 20 μm.

The localization of the MgGPP protein (5 dpi) was consistently observed in giant cell nuclei. In some sections, MgGPP was also observed along the cell wall of adjacent giant cells around the nematode head or accumulated on the nematode head, at the stylet and inside the lumen of the anterior esophagus (Fig 2). No signal was observed in the giant cells of either gall sections containing nematodes without hybridization or incubated with pre-immune serum or in root sections of an uninfected healthy plant hybridized with anti-MgGPP serum (S4 Fig). These findings suggested that MgGPP is secreted in the early stages of *M*. *graminicola* parasitism, injected into the root tissue, and targeted to the host cell nuclei.

### Relocation of MgGPP from the ER to the nucleus and its glycosylation and proteolysis in host cells

**Fig 3:**
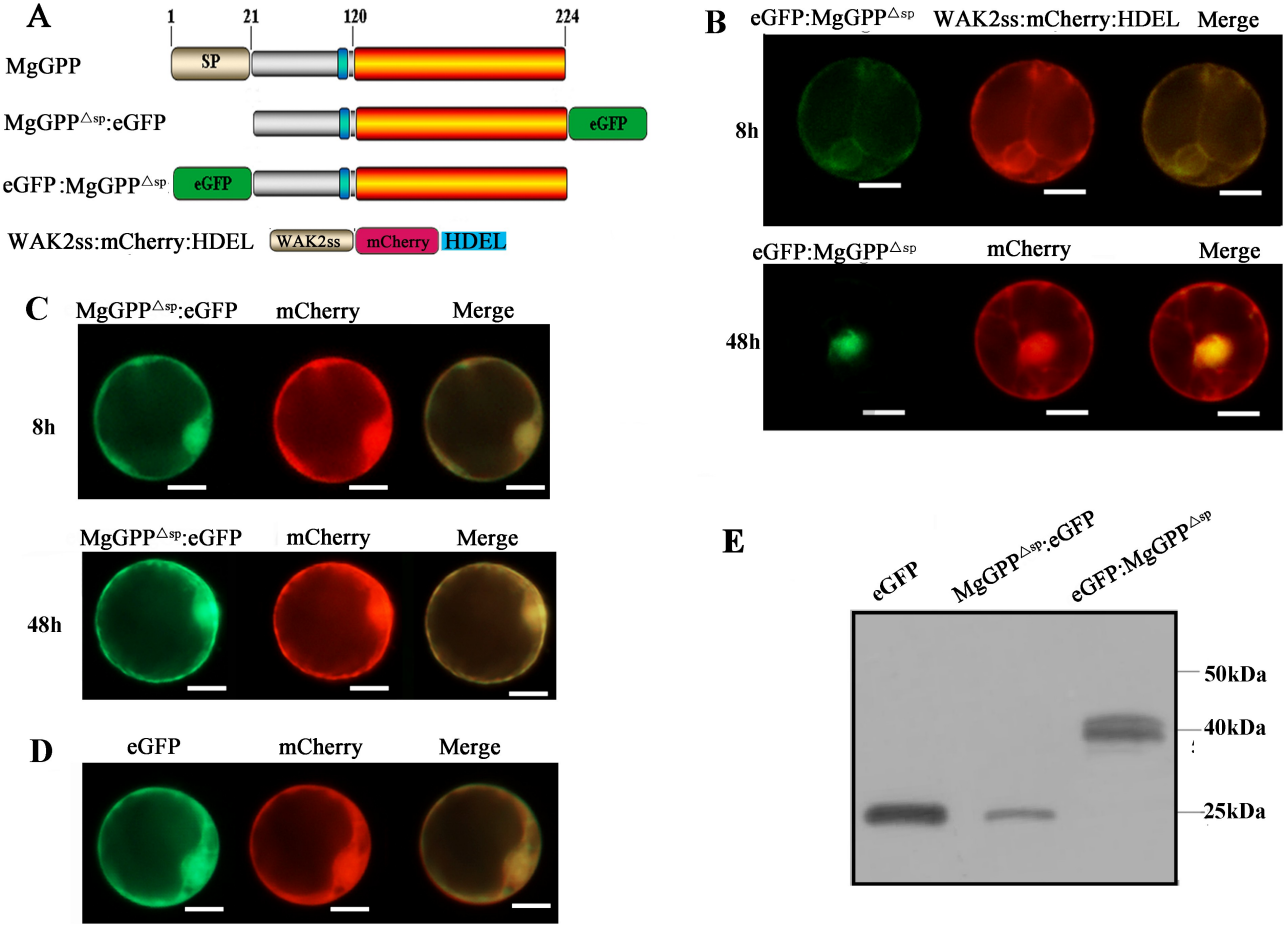
Subcellular localization of MgGPP in the rice root protoplast cells. (A) Schematic diagram showing the fusion protein structures of MgGPP. (B) eGFP:MgGPP^Δsp^ was transformed into rice root protoplasts. HDEL is a signal for retention in the ER, and WAK2ss-mCherry-HDEL was used as a marker to indicate the ER. The NH2-terminal signal sequence (WAK2ss) from *Arabidopsis thaliana* wall-associated kinase 2 was used to direct the fusion protein to secretory compartments. Signals that colocalized with the ER marker WAK2ss-mCherry-HDEL were observed in the ER at ~8 h after cotransformation, and signals that colocalized with mCherry were observed in the nuclei at ~48 h after cotransformation. (C) MgGPP^Δsp^:eGFP was transformed into rice root protoplasts. Signals that colocalized with mCherry were observed in the cytoplasm and nuclei at ~8 h and ~48 h after cotransformation. (D) Free eGFP was transformed into rice root protoplasts. Signals that colocalized with mCherry were observed in the whole transformed cells. (E) Using an anti-GFP antibody, western blot analysis of proteins from transformed cells showed the ~27 kDa size of MgGPP^Δsp^:eGFP, which was much smaller than the expected size of MgGPP^Δsp^:eGFP (~50 kDa) but identical to the size of eGFP, and two protein forms of ~43 and ~39 kDa of eGFP:MgGPP^Δsp^ that were both smaller than the expected size of eGFP:MgGPP^Δsp^ (~50 kDa). These indicated that MgGPP may be processed and cleaved. Scale bar, 50 μm.

To intensively study the subcellular localization of MgGPP in host cells, a transient protein expression assay was performed using protoplasts from rice roots. The full-length MgGPP sequence without the signal peptide region was fused with enhanced green fluorescent protein (eGFP) and transformed into rice root protoplasts. The eGFP was fused to either the N-terminus (eGFP:MgGPP^Δsp^) or C-terminus (MgGPP^Δsp^:eGFP) of MgGPP (Fig 3A). Since MgGPP was confirmed to be secreted inside host cells, the exclusion of the signal peptide should allow the MgGPP effector to be tested for its function in host cells. At ~8 h after culture, the fusion protein eGFP:MgGPP^Δsp^ was detected to be colocalized in the ER with the ER marker HDEL in ~41% of the transformed cells. After ~48 h, the exclusive nuclear localization of eGFP:MgGPP^Δsp^ in ~42% of the transformed cells was detected (Fig 3B). Unexpectedly, cells transformed with the fusion protein MgGPP^Δsp^:eGFP consistently displayed both cytoplasmic and nuclear accumulation of the fluorescent signal (Fig 3C). As a control, the transformed cells expressing eGFP alone also showed cytoplasmic and nuclear accumulation of the GFP signal (Fig 3D).

To demonstrate the correct expression of eGFP-tagged MgGPP in protoplast cells, the proteins extracted from the transformed cells were analyzed by western blot using an anti-GFP antibody. Unexpectedly, when MgGPP^Δsp^:eGFP was transiently expressed in protoplasts from rice roots, the anti-GFP antibody specifically detected an accumulated protein of ~27 kDa, which was much smaller than the expected size of MgGPP^Δsp^:eGFP (~50 kDa) but identical to the size of eGFP, indicating that the C- terminus of MgGPP may be proteolytically cleaved, and the fluorescence in the cytoplasm and nucleus could be the free eGFP. In contrast, when eGFP:MgGPP^Δsp^ was transiently expressed in protoplasts, two protein forms of ~43 and ~39 kDa were detected, which was one extra protein form than we expected. Moreover, they were both smaller than the expected size of eGFP:MgGPP^Δsp^ (~50 kDa) (Fig 3E), indicating that MgGPP may be processed and cleaved.

**Fig 4:**
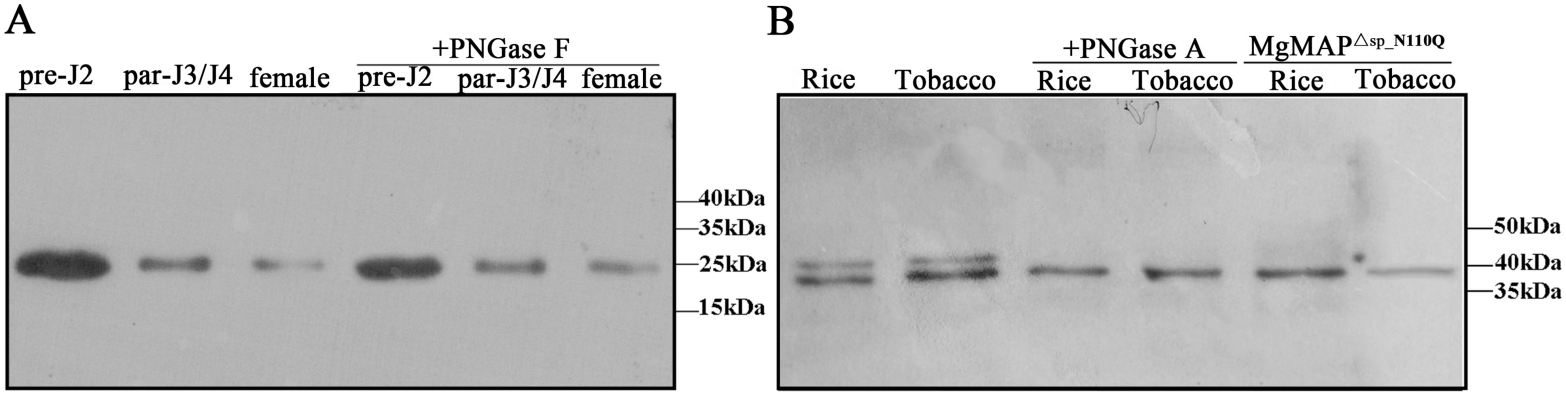
Assays for glycosylation of MgGPP. (A) Using an anti-MgGPP antibody, western blot analysis of proteins from pre-J2s, par-J3s/J4s and females of *Meloidogyne graminicola* treated with or without PNGase F all showed the ~25 kDa size band, indicating that MgGPP is not glycosylated in nematodes. (B) Using an anti-GFP antibody, western blot analysis of proteins from the transformed cells of rice and tobacco showed two protein forms of ~43 and ~39 kDa of eGFP:MgGPP^Δsp^, the ~39 kDa size of MgGPP^Δsp^:eGFP treated with PNGase A, and ~39 kDa size of the point mutation eGFP:MgGPP^Δsp_N110Q^, indicating that *N*-glycosylation of MgGPP occurred in host plants.

*In silico* analysis showed that MgGPP has one *N*-glycosylation site at Asn-110, showing that MgGPP may be glycosylated. An anti-MgGPP antibody specifically detected a band with a size of ~25.5 kDa in the total protein samples from pre-J2s, par-J3s/J4s and females with or without treatment of the deglycosylation enzyme PNGase F (Fig 4A), indicating that MgGPP is not glycosylated in nematodes. Therefore, we speculated that the host plants mediated the *N*-glycosylation and *C*-proteolysis of MgGPP. To verify this speculation, first, total protein samples of rice protoplasts and tobacco leaves expressing eGFP:MgGPP^Δsp^ were treated with the deglycosylation enzyme PNGase A. Western blot analysis showed that the protein form of ~43 kDa was successfully cleaved by PNGase A (Fig 4B). Second, we also generated an MgGPP allele in which the N110Q site was mutated and expressed this mutant in rice protoplasts and tobacco leaves. Western blotting analysis showed that the protein form of ~43 kDa also disappeared (Fig 4B). Based on these results, we conclude that the secreted effector protein MgGPP is *N*-glycosylated in host cells.

**Fig 5:**
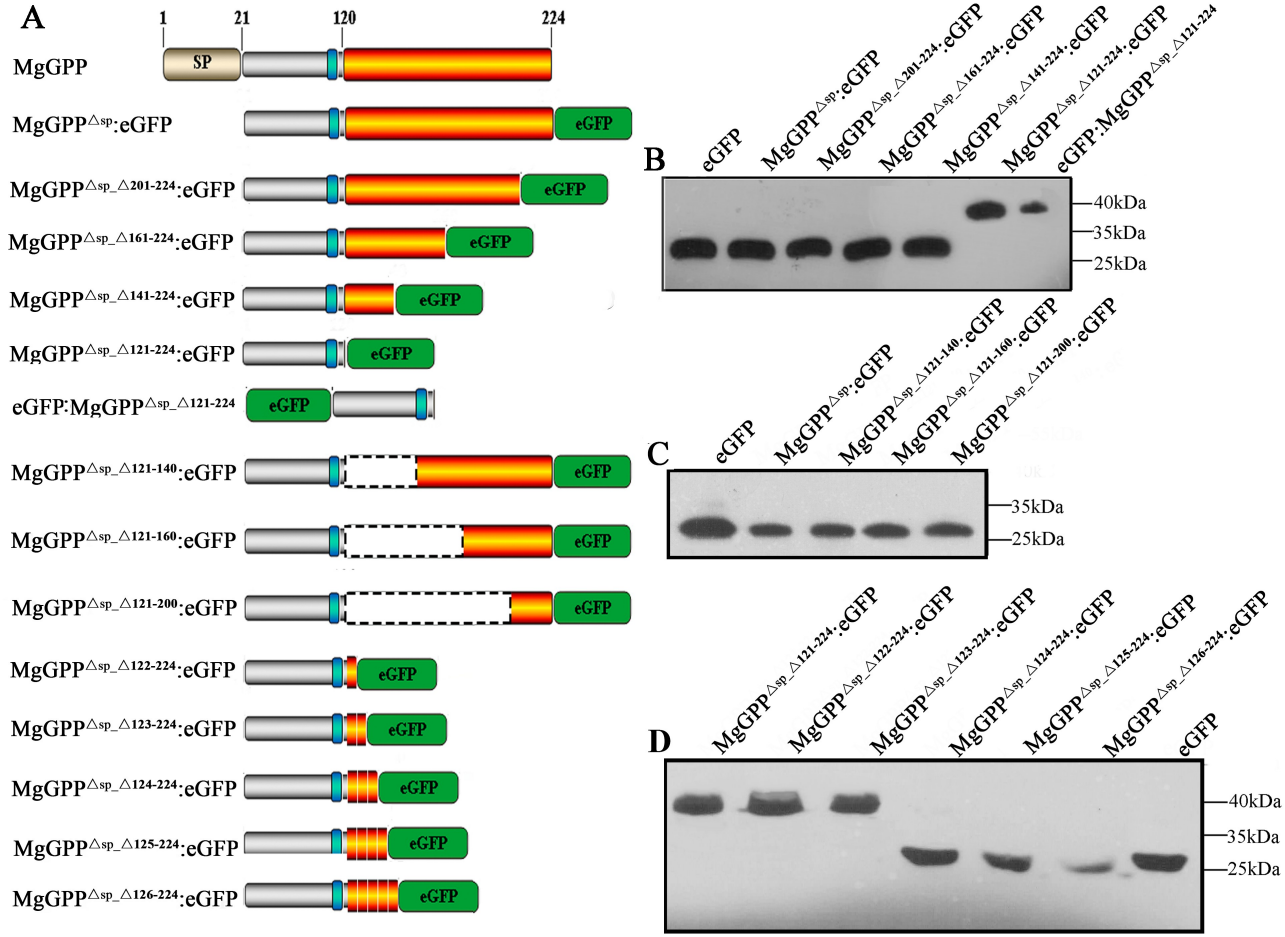
MgGPP^123-224^ is processed proteolytically in multiple loci. (A) Schematic diagram showing the protein structures of MgGPP mutants and the bluish bar represents the glycosylation site (N110). (B) Using an anti-GFP antibody, western blot analysis of proteins from transformed cells showed the ~27 kDa size of MgGPP^Δsp_Δ201–224^:eGFP, MgGPP^Δsp_Δ161–224^:eGFP and MgGPP^Δsp_Δ141–224^:eGFP, which were identical to the size of eGFP, and the ~39 kDa size of MgGPP^Δsp_Δ121–224^:eGFP that was identical to the size of eGFP:MgGPP^Δsp_Δ121–224.^ (C) Using an anti-GFP antibody, western blot analysis of proteins from transformed cells all showed the ~27 kDa size of MgGPP^Δsp_Δ121–140^:eGFP, MgGPP^Δsp_Δ121–160^:eGFP and MgGPP^Δsp_Δ121–200^:eGFP that were identical to the size of eGFP. (D) Using an anti-GFP antibody, western blot analysis of proteins from transformed cells showed the ~39 kDa size of MgGPP^Δsp_Δ121–224^:eGFP, MgGPP^Δsp_Δ122–224^:eGFP and MgGPP^Δsp_Δ123–224^:eGFP, and ~27 kDa size of MgGPP^Δsp_Δ124–224^:eGFP, MgGPP^Δsp_Δ125–224^:eGFP and MgGPP^Δsp_Δ126–224^:eGFP that were identical to the size of eGFP. These indicated that MgGPP was cleaved in multiple loci from 123 to 224 aa.

Because MgGPP may be subjected to *C*-proteolysis, our original goal was to determine the cleavage site in MgGPP. To achieve this, MgGPP^Δsp_Δ201–224^:eGFP, MgGPP^Δsp_Δ161–224^:eGFP, MgGPP^Δsp_Δ141–224^:eGFP and MgGPP^Δsp_Δ121–224^:eGFP (Fig 5A) were generated and transiently expressed in rice protoplasts. Western blot analysis indicated that the anti-GFP antibody specifically detected a band with the size of ~27 kDa, which is identical to the size of free eGFP, in plants expressing MgGPP^Δsp_Δ201–224^:eGFP, MgGPP^Δsp_Δ161–224^:eGFP and MgGPP^Δsp_Δ141–224^:eGFP. However, plants expressing MgGPP^Δsp_Δ121–224^:eGFP produced a ~39 kDa band that corresponded to the molecular weight of the fusion protein of eGFP plus MgGPP^Δsp_Δ121–224^ (Fig 5B). Furthermore, MgGPP^Δsp_Δ121–140^:eGFP, MgGPP^Δsp_Δ121–160^:eGFP and MgGPP^Δsp_Δ121–200^:eGFP (Fig 5A) were constructed and transiently expressed in rice protoplasts. Similarly, the anti-GFP antibody specifically detected a band of ~27 kDa that is the same size as free eGFP (Fig 5C). These results suggested that MgGPP was cleaved at multiple loci from 121 to 224 aa, and the cleavage position nearest to the N-terminus should be between 121 aa and 140 aa. Then, MgGPP^Δsp_Δ122–224^:eGFP, MgGPP^Δsp_Δ123–224^:eGFP, MgGPP^Δsp_Δ124–224^:eGFP, MgGPP^Δsp_Δ125–224^:eGFP and MgGPP^Δsp_Δ126–224^:eGFP were constructed (Fig 5A) and transiently expressed in rice protoplasts. Western blot analysis showed that the expression of MgGPP^Δsp_Δ122–224^:eGFP and MgGPP^Δsp_Δ123–224^:eGFP generated a ~39 kDa band, but the expression of MgGPP^Δsp_Δ124–224^:eGFP, MgGPP^Δsp_Δ125–224^:eGFP and MgGPP^Δsp_Δ126–224^:eGFP produced a ~27 kDa band (Fig 5D). These results showed that the secreted effector protein MgGPP is processed proteolytically after the 122-aa position in host cells.

### MgGPP^123-224^ region is required for MgGPP trafficking to the ER

**Fig 6:**
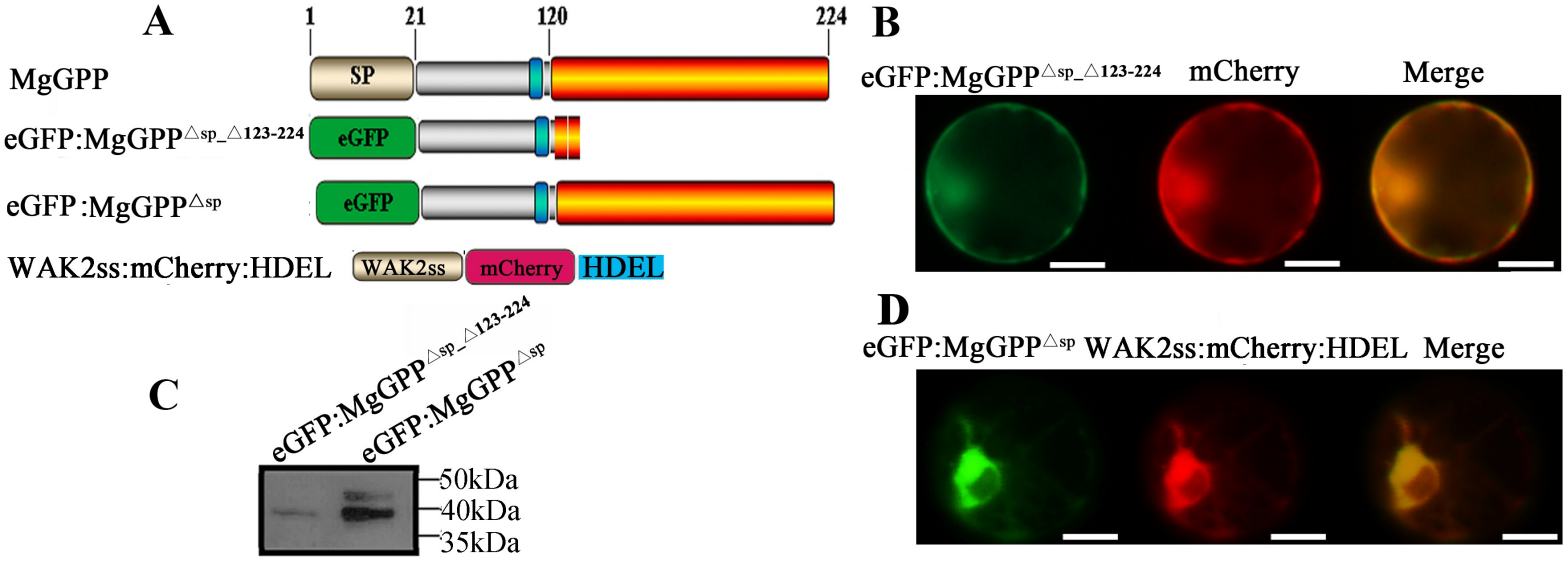
Subcellular localization of MgGPP^Δsp_Δ123–224^ in the rice root protoplast cells. (A) Schematic diagram showing the protein structures of MgGPP mutants. (B) eGFP:MgGPP^Δsp_Δ123–224^ was transformed into rice root protoplasts. Signals that colocalized with mCherry were observed in the cytoplasm and nuclei at ~8 h after cotransformation. (C) Using an anti-GFP antibody, western blot analysis of proteins from transformed cells showed a ~39-kDa band in cells transformed with eGFP:MgGPP^Δsp_Δ123–224^ and two protein forms of ~43 and ~39 kDa in the cells transformed with eGFP:MgGPP^Δsp^. (D) As a control, eGFP:MgGPP^Δsp^ was transformed into rice root protoplasts. Signals were observed in the ER at ~8 h after cotransformation. These indicated MgGPP lacking amino acids 123–224 could not be imported into the ER and glycosylated. Scale bar, 50 μm.

The 123-aa to 224-aa C-terminal sequence of MgGPP is processed proteolytically when MgGPP enters host plant cells. Additionally, the analysis of the subcellular localization of MgGPP showed that MgGPP translocated from the ER to the nucleus. It has been considered that proteolytic processing may function in the proper trafficking of effectors to their cellular targets [32], and we therefore speculated that the MgGPP^123-224^ region may be required for MgGPP trafficking to the ER. As mentioned above, the 123-aa to 224-aa C-terminal sequence of MgGPP is processed proteolytically, which motivated us to study the role of this region. Thus, eGFP:MgGPP^Δsp_Δ123–224^ was generated (Fig 6A) and transiently expressed in rice protoplasts. The results show that no ER localization signal was observed in any transformed cells at ~8 h after culture, while the fluorescent signal was observed in both the nucleus and cytoplasm (Fig 6B). Moreover, the anti-GFP antibody specifically detected an accumulated protein of ~39 kDa (Fig 6C). As a control, nearly half of the eGFP:MgGPP^Δsp^ was observed in the ER (Fig 6D). These results indicated that MgGPP lacking the sequence of 123 aa to 224 aa could not be imported into the ER and glycosylated.

**Fig 7:**
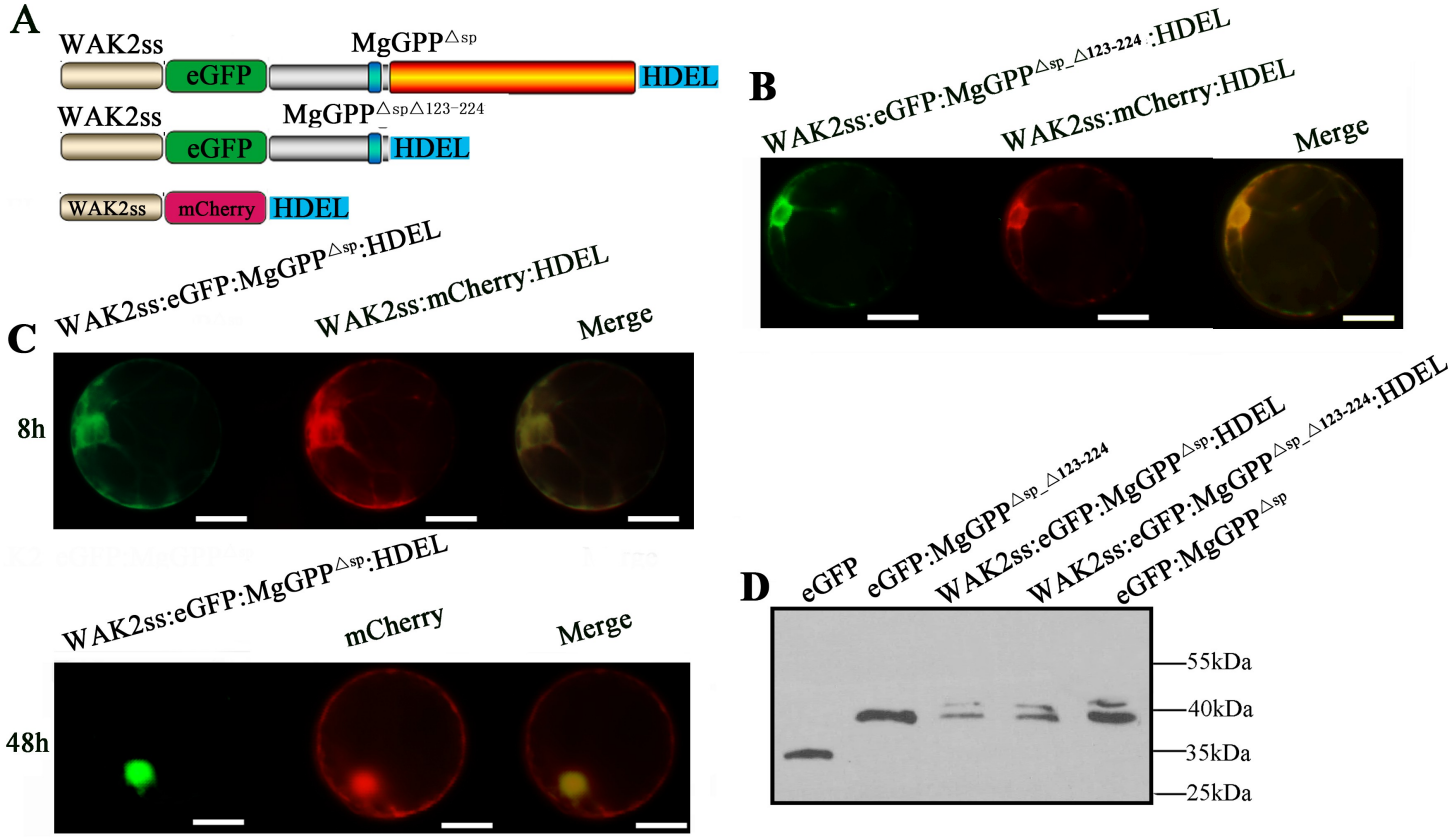
Subcellular localization of eGFP:MgGPP^Δsp_Δ123–224^:HDEL and eGFP:MgGPP^Δsp^:HDEL in the rice root protoplast cells. (A) Schematic diagram showing the protein structures of WAK2ss:eGFP:MgGPP^Δsp_Δ123–224^:HDEL and WAK2ss:eGFP:MgGPP^Δsp^:HDEL. (B) WAK2ss:eGFP:MgGPP^Δsp_Δ123–224^:HDEL was transformed into rice root protoplasts. HDEL is a signal for retention in the ER, and WAK2ss-mCherry-HDEL was used as a marker to indicate the ER. The NH2-terminal signal sequence (WAK2ss) from *Arabidopsis thaliana* wall-associated kinase 2 was used to direct fusion proteins to secretory compartments. Signals that colocalized with the ER marker WAK2ss-mCherry-HDEL were consistently observed in the ER after cotransformation. (C) WAK2ss:eGFP:MgGPP^Δsp^:HDEL was transformed into rice root protoplasts. Signals that colocalized with the ER marker WAK2ss-mCherry-HDEL were observed in the ER at ~8 h after cotransformation, and signals that colocalized with mCherry were observed in the nuclei at ~48 h after cotransformation. (D) Using an anti-GFP antibody, western blot analysis of proteins from transformed cells showed two protein forms of ~43 and ~39 kDa of both eGFP:MgGPP^Δsp_Δ123–224^:HDEL and eGFP:MgGPP^Δsp^:HDEL. These indicated that the glycosylation of MgGPP occurred in the ER, and proteolysis of MgGPP^123-224^ led MgGPP^Δsp_Δ123–224^ to leave the ER. Scale bar, 50 μm.

*N*-Glycosylation of proteins in eukaryotic cells usually occurs in the ER [33]. We therefore speculated that the MgGPP^123-224^ region is required for ER import of MgGPP, resulting in the glycosylation of MgGPP in the ER. To verify this, we constructed WAK2ss:eGFP:MgGPP^Δsp^:HDEL and WAK2ss:eGFP:MgGPP^Δsp_Δ123–224^:HDEL (Fig 7A) to ensure that MgGPP was transported to and retained in the ER. When the two vectors were transiently expressed in rice protoplasts, the fluorescent signal of WAK2ss:eGFP:MgGPP^Δsp_Δ123–224^:HDEL was consistently observed in the ER (Fig 7B), and WAK2ss:eGFP:MgGPP^Δsp^:HDEL was observed in the ER of ~50% of the transformed cells at ~8 h after culture and in the nucleus of ~40% of the transformed cells after ~48 h (Fig 7C). The results demonstrated that the proteolytic cleavage of MgGPP at site 123 releases MgGPP^21-122^ from the C-terminal part containing the ER-retention signal, and therefore this amino-terminal part can leave the ER. Western blot analysis showed that two bands of ~43 and ~39 kDa were detected, indicating that these two fusion proteins were both glycosylated in the ER (Fig 7D). Thus, we conclude that the MgGPP^123-224^ region is required for MgGPP trafficking to the ER, and the effector is then glycosylated in the ER.

### MgGPP affects *M*. *graminicola* parasitism

**Fig 8:**
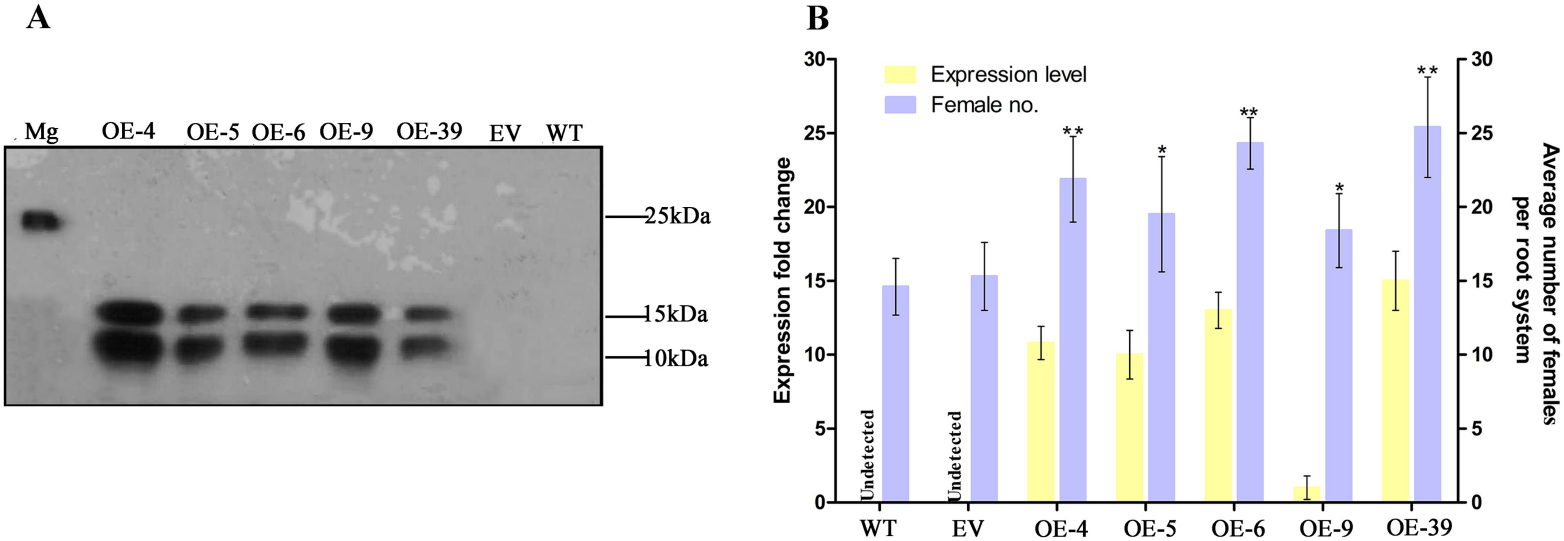
Transgenic lines expressing MgGPP in rice exhibit enhanced susceptibility to *Meloidogyne graminicola*. (A) Western blot confirmation of the MgGPP product with an anti-MgGPP antibody. Two protein forms of ~12 and ~ 16 kDa were detected because of the glycosylation and proteolysis of MgGPP. As a control, one protein of ~25 kDa was detected in *M*. *graminicola*. (B) qRT-PCR analysis was used to confirm the *MgGPP* mRNA expression level in transgenic-*MgGPP* lines. Transgenic rice expressing *MgGPP* showed an increased number of females in roots compared with the controls. The data are presented as the means ± standard deviation (SD) from fifteen plants. *P < 0.05; **P < 0.01, Student’s t test. OE-4, 5, 6, 9 and 39, five transgenic rice lines; Mg, *M*. *graminicola*; EV, empty vector; WT, wild type.

To assess the role of MgGPP in nematode parasitism, transgenic rice lines expressing MgGPP without the signal peptide under the control of the maize ubiquitin promoter were generated. Southern blot analysis confirmed the integration of the target gene into the rice genome, and five single-copy transgenic lines were selected (S5A Fig). The expression of MgGPP transcripts in the five transgenic lines was confirmed by qRT-PCR analysis (S5B Fig). Meanwhile, western blot analysis was also used to examine the expression of MgGPP using the anti-MgGPP antibody (Fig 8A). The western blot analysis showed that two bands of ~12 kDa and ~16 kDa were detected, which is consistent with the expected molecular weight of the protein, due to PTM of MgGPP in rice plants. MgGPP expression seemed to have no measurable impact on transgenic plant growth and development by phenotype analysis. Then, the susceptibility of these transgenic rice lines to nematodes was tested. The results indicated that the average number of adult females increased by 49.8% and 42.9%, 33.6% and 27.5%, 62.2% and 58.8%, 26% and 21.3%, and 73% and 66% at 12 dpi in lines 4, 5, 6, 9 and 39, respectively, compared to the wild-type (WT) and empty vector (EV) plants. In conclusion, transgenic rice lines overexpressing MgGPP were more susceptible to nematode infection (Fig 8B).

**Fig 9:**
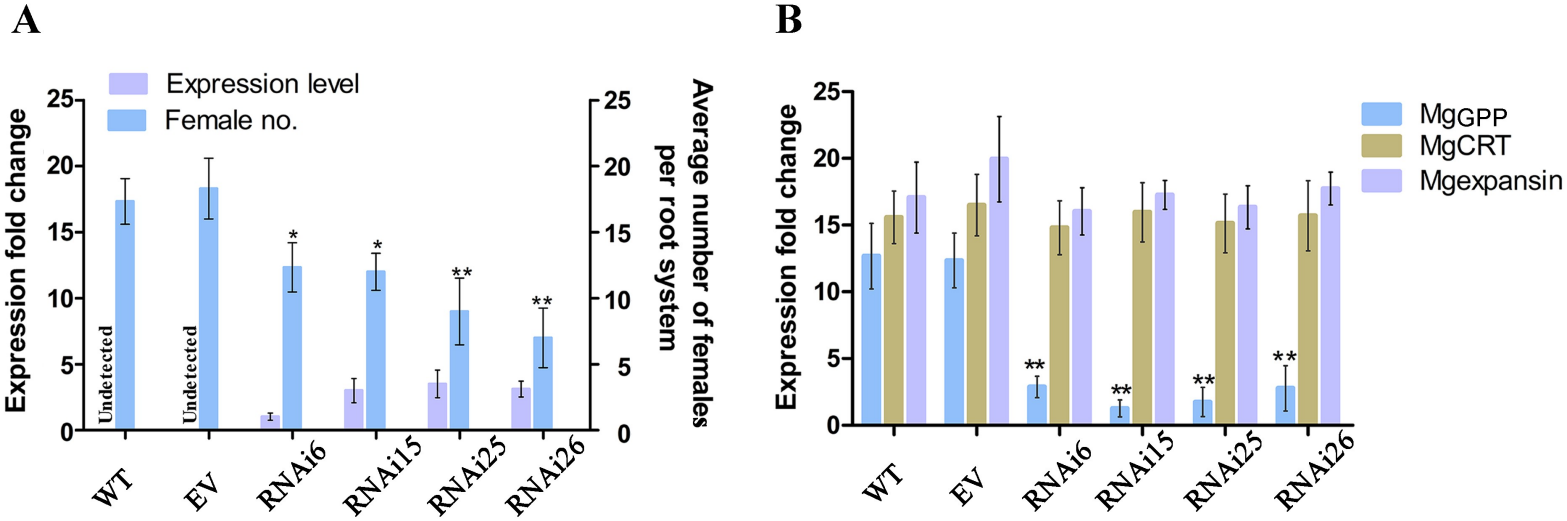
*In planta* RNAi of *MgGPP* attenuates *Meloidogyne graminicola* parasitism. (A) qRT-PCR analysis to detect the GUS intron fragment was used to confirm dsRNA expression levels in roots of RNAi lines. Transgenic RNAi rice lines showed a decreased number of females in roots compared with the controls. The data are presented as the means ± standard deviation (SD) from fifteen plants. (B) qRT-PCR assays of the expression levels of *MgGPP* in *M*. *graminicola* collected from RNAi lines, transgenic empty vector (EV) plants and wild type (WT) plants. The expression levels of *Mg-CRT* and *Mg-expansin* from *M*. *graminicola* were used to determine the specificity of the *MgGPP-*targeting RNAi. *P < 0.05; **P < 0.01, Student’s t test. RNAi6, 15, 25 and 26, different transgenic RNAi rice lines.

To further confirm the findings of *MgGPP* overexpression, host-mediated gene silencing was performed to knock down *MgGPP* expression during the parasitism of *M*. *graminicola* using transgenic rice lines expressing a hairpin dsRNA of *MgGPP*. Four single-copy transgenic rice plants were confirmed using southern blot analysis (S5C Fig). By RT-PCR and qRT-PCR, these transgenic plants were confirmed to carry the MgGPP dsRNA, that is, the RNAi cassette (a 363-bp GUS intron fragment) was detected in the four single-copy transgenic lines (S5D Fig and Fig 9A) and was not amplified in the WT and EV controls. According to phenotype analyses, the transgenic lines expressing the hairpin dsRNA of MgGPP had no apparent differences in plant growth and development compared to the WT control lines. The expression level of *MgGPP* in nematodes after silencing by host-mediated RNAi was measured by qRT-PCR. The transcription of MgGPP was reduced significantly in *M*. *graminicola* feeding on the roots of RNAi lines at 3 dpi compared to those feeding on control plants. Therefore, the host-mediated gene silencing of *MgGPP* was effective. Two other genes, *Mg-CRT* and *Mg-expansin*, which have similar transcriptional expression patterns and somewhat similar nucleotide sequences to those of *MgGPP*, were used to verify the specificity of this *MgGPP*-targeting RNAi by qRT-PCR analysis. The results showed that *Mg-CRT* and *Mg-expansin* were not affected by the *MgGPP*-targeting RNAi treatment (Fig 9B). Importantly, the four transgenic rice lines had 50%-72.2% fewer adult females than the WT lines and EV plants at 12 dpi (Fig 9A). These findings suggest that *MgGPP* plays a role in nematode parasitism.

### Glycosylated MgGPP protein can suppress cell death induced by Gpa2/RBP-1

**Fig 10:**
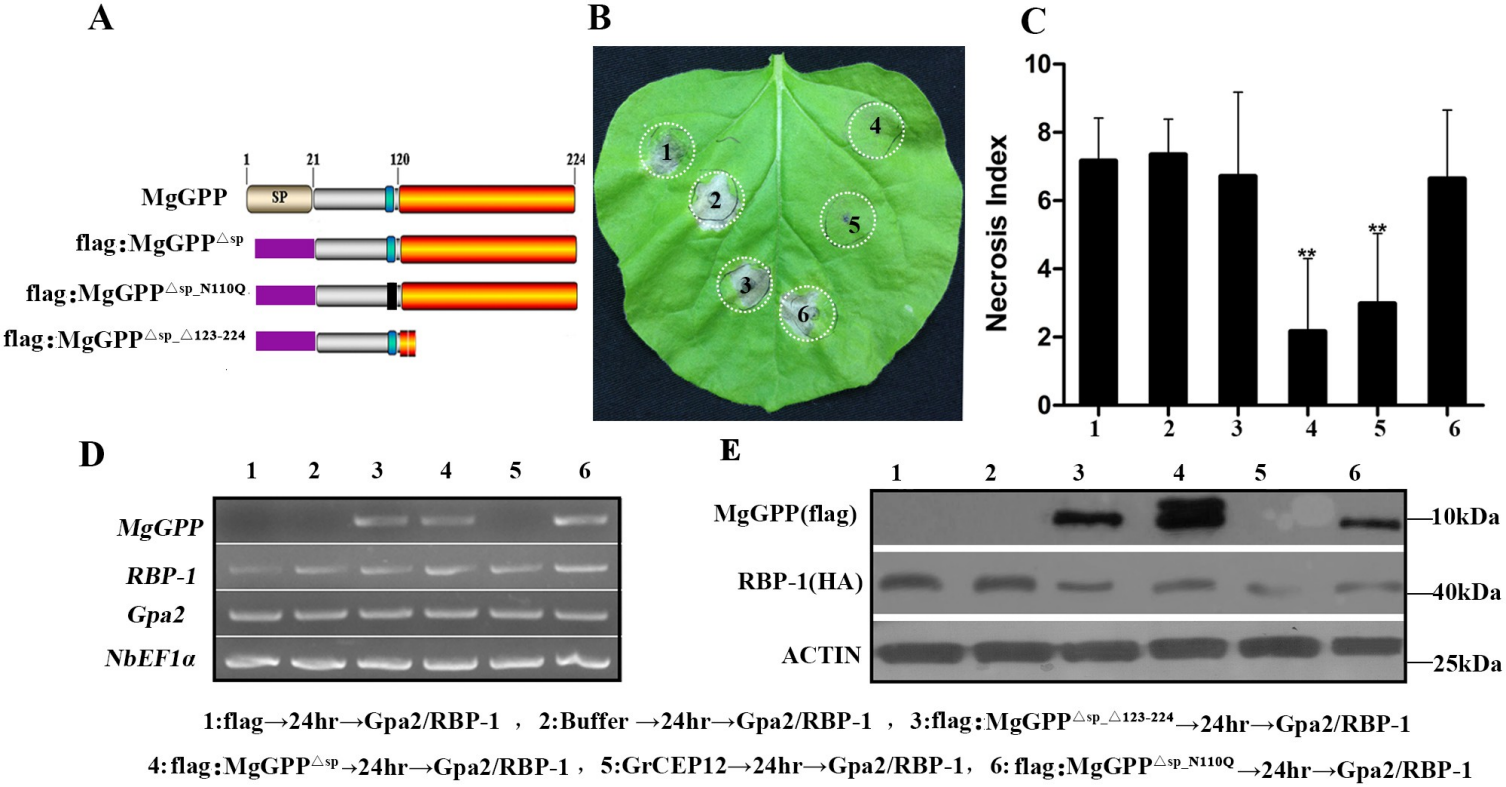
Suppression of Gpa2/RBP-1-triggered cell death by MgGPP. (A) Schematic diagram showing the protein structures of MgGPP mutants. (B) Assay of the suppression of Gpa2/RBP-1**-**triggered cell death in *Nicotiana benthamiana* by MgGPP. *N*. *benthamiana* leaves were infiltrated with buffer or *Agrobacterium tumefaciens* cells carrying flag:MgGPP^Δsp_Δ123–224^, flag:MgGPP^Δsp^, GrCEP12, flag:MgGPP^Δsp_N110Q^ and the flag control gene, followed 24 h later with *A*. *tumefaciens* cells carrying the Gpa2/RBP-1 genes. The cell death phenotype was scored and photographs were taken 5 days after the last infiltration. (C) The average areas of cell death of in leaves infected with MgGPP and other proteins followed by Gpa2/RBP-1. The columns with asterisks indicates a highly statistically significant reduction of the necrosis index of MgGPP and GrCEP12 compared with that of the negative control flag. Each column represents the mean with the standard deviation (n = 55). **P<0.01, Student’s t test. (D) RT-PCR confirmation of the expression of *MgGPP*, *RBP-1* and *Gpa2*. (E) Western blot analysis was used to confirm the expression of RBP-1, MgGPP and MgGPP mutants with an anti-GFP antibody.

There is evidence that secreted effectors originating from the nematode esophageal gland cells can directly suppress the plant defense system that is responsible for the parasitism of nematodes [9,10,12,16,34]. Bax, INF1 and Gpa2/RBP-1 can trigger cell death [9,12,35]; therefore, to determine whether MgGPP actually has the ability to suppress the plant immune system, Bax, INF1 and Gpa2/RBP-1 systems were used to investigate the function of MgGPP in programmed cell death (PCD). *Agrobacterium* strains carrying flag:MgGPP^Δsp^ were constructed (Fig 10A). In addition, the empty vector pCAMBIA1305:flag was generated as a negative control, and pCAMBIA1305:GrCEP12 that can suppress the HR induced by Gpa2/RBP-1 [12] was used as the positive control. These constructs were infiltrated into *Nicotiana benthamiana* leaves 24 h prior to infiltration of an *Agrobacterium* strain carrying Bax, INF1 and Gpa2/RBP-1. At 5 days after the last infiltration, we found that MgGPP and the positive control GrCEP12 could suppress cell death mediated by Gpa2/RBP-1, with a necrosis index of 2.6 and 3.0, respectively. As controls, all points infiltrated with buffer and flag followed by Gpa2/RBP-1 showed a necrosis index of around 7 (Fig 10B and 10C), whereas infiltration with buffer, MgGPP or flag alone did not induce necrosis (S6 Fig). In addition, none of the treatments, including infiltration of MgGPP, could suppress the HR induced by Bax and INF1 (S6 Fig).

We have shown above that MgGPP is subjected to *N*-glycosylation and *C*-proteolysis in plants, and the MgGPP^123-224^ region is required for MgGPP trafficking to the ER, where MgGPP is glycosylated. The glycosylation of effectors may suppress plant immunity [19]. To discover if glycosylation of MgGPP affected its ability to suppress the HR, we constructed the pCAMBIA:flag:MgGPP^Δsp_Δ123–224^ and pCAMBIA:flag:MgGPP^Δsp-N110Q^ mutants to generate non-glycosylated MgGPP (Fig 10A). It was found that all points infiltrated with pCAMBIA:flag:MgGPP^Δsp_Δ123–224^ and pCAMBIA:flag:MgGPP^Δsp-N110Q^ followed by Gpa2/RBP-1 showed necrosis (Fig 10B and 10C). The expression of all genes was verified by RT-PCR (Fig 10D) and western blot analysis (Fig 10E). These results showed that the glycosylated MgGPP protein can suppress the cell death induced by Gpa2/RBP-1.

## Discussion

In this study, our original aim was to amplify a *Meloidogyne* avirulence proteins (MAPs) gene based on contigs that were annotated as *Map* from a previously reported transcriptome of *M*. *graminicola* [29]. Although the amplified gene exhibited the highest match with MAPs, the shared identities were no more than 41.1%. Moreover, this gene does not possess a conserved RlpA-like protein domain and internal repetitive motifs, which are common in MAPs [36,37]. These observations showed that the gene is not the counterpart of the reported *Map* genes of *Meloidogyne* and is a novel gene that we have called *MgGPP*.

Some evidence from this study indicated that the novel nematode effector MgGPP is secreted into host plants and plays a role in nematode parasitism. First, *in silico* analysis demonstrated that MgGPP contains an N-terminal signal peptide, which is considered to be a character of secreted proteins [38]. In addition, *in situ* hybridization indicated that *MgGPP* was expressed exclusively in the subventral esophageal glands, which are one of the origins of nematode secretory effector proteins and are thought to be involved in the early parasitic stages of RKNs [5]. Second, qRT-PCR analysis showed that the transcription of MgGPP was obviously up-regulated during the early parasitic stages of the nematodes, reaching a maximum level at 3 dpi, which is consistent with the results of its spatial expression, suggesting a potential role in the early parasitism stage of nematodes. Third, we affirmed, using an immunocytochemical method, that MgGPP accumulated in host giant cell nuclei, showing that MgGPP could indeed be secreted into host plant cells. Fourth, rice transgenic lines overexpressing *MgGPP* became substantially more susceptible to the nematode infection than wild-type plant controls, and conversely, silencing of *MgGPP* using an *in planta* RNAi approach significantly attenuated nematode parasitism, demonstrating that MgGPP promoted *M*. *graminicola* parasitism.

The identification of the subcellular compartments targeted by nematode-secreted effectors could assist in their functional characterization [39]. MgGPP was found to contain one NLS in the N-terminus. It is usually considered that nematode effectors with one or more NLS are most likely to be a nuclear-localized protein [38,40,41]. However, it has also been reported that nematode effectors possessing NLSs were not located in the nucleus [39]. The immunocytochemical technique is a good tool for studying the actual localization of nematode-secreted effectors *in planta* [42]. Two effectors, Mi-EEF1 and MjNULG1a, secreted by *M*. *incognita* and *M*. *javanica*, respectively, both containing NLSs, were confirmed to be nuclear-localized using the immunocytochemical method [40,41]. Utilizing this technique, we confirmed that MgGPP was actually targeted to giant cell nuclei. Meanwhile, it was noticed that the MgGPP signal was also observed in the cell-wall regions around the head of the nematode. It was reported that the stylet comes into contact with the plasma membrane without perforation and delivers nematode-secreted proteins in the cytoplasm [43]. Two RKNs-secreted effectors have been observed in the apoplast and to target to the nuclei [40,41], raising the possibility that PPNs effectors could be translocated from the apoplasm to the cytoplasm of plant cells., although little is known about transport mechanisms of PPNs effectors. Interestingly, the effectors from oomycetes and fungi were shown capable of further moving into plant cells after entering the apoplast [44]. For example, the oomycete effectors’ RXLR motif mediated entry of the effectors into cells by binding to the phospholipid, phosphatidylinositol-3-phosphate that is abundant on the outer surface of plant cell plasmamembranes. Therefore, it is thought that RxLR effector entry involves lipid raft-mediated endocytosis [45,46]. Regardless of how the effector MgGPP enters plant cells from the apoplast, our results suggest that MgGPP is probably secreted by the nematode into the apoplast, then entering into cells and targeting into the cell nucleus during the process of nematode parasitism. However, it is interesting that transiently expressed MgGPP was not always located in the nucleus. At ~8 h after culture, MgGPP can be observed in the ER of rice root protoplasts, and finally in the nucleus at ~48 h. With all these considered, it is possible that MgGPP may be secreted into the plant cell apoplast first and then enter into host cells and target to the ER, before finally being transported to the nucleus.

In this study, most importantly, MgGPP was found to be post-translationally modified in host plants. Thus far, only three nematode-secreted effectors have been found to be post-translationally modified, including GrUBCEP12 and CLE from *G*. *rostochiensis* and 10A07 from *H*. *schachtii*, which were proteolytically cleaved, glycosylated and phosphorylated *in planta*, respectively [12,24,25]. Detailed studies have shown that MgGPP was subjected to both *N*-glycosylation and C-terminal proteolysis in host cells. Recent studies showed that glycosylation is one of the pathogen effector PTMs [47,48]. More often, the glycosylation of effectors occurred in the plant pathogen itself, and the subsequent glycoprotein was secreted into host plants. For example, the secreted effectors BAS4, CBH1 and PCIPGII were shown to undergo *N*-glycosylation in *M*. *oryzae*, *Trichoderma reesei* and *Phytophthora capsici*, respectively, and then translocate into the host cytoplasm [19,49–51]. Only a few effectors have been found to be first secreted into host plants and then glycosylated in the plants, such as the CLE effector from *G*. *rostochiensis* mentioned above [25].

Previous reports indicated that the glycosylation of effectors may be related to plant immunity [19]. For example, *N*-glycosylation of the effector Slp1 of *M*. *oryzae* enhanced its ability to suppress the host immunity by binding chitin oligosaccharides [19,52], while *N*-glycosylation of the effectors Avr4 and Avr9 of *Cladosporium fulvum* improved their capability to induce effector-triggered defense responses [53]. In this study, we confirmed that *N*-glycosylated MgGPP can consistently suppress the cell death induced by effector-triggered immunity (ETI) proteins Gpa2/RBP-1, while non-*N*-glycosylated MgGPP cannot. Additionally, neither *N*-glycosylated MgGPP nor non-*N*-glycosylated MgGPP can suppress the cell death induced by the PAMP-triggered immunity (PTI) protein INF1 and the pro-apoptotic protein Bax. Consistent with this notion, many plant pathogen effectors have been shown to selectively suppress the host cell death responses induced by several elicitors. For example, allergen-like proteins of cyst nematodes selectively suppress the activation of programmed cell death by surface-localized immune receptors, and different effectors of *Phytophthora sojae* could selectively suppress the programmed cell death induced by different elicitors [34,54]. Thus, it seems that glycosylation of MgGPP contributed to the its ability to selectively suppress the defense-related host cell death, which is one possible mechanism underlying its contribution to *M*. *graminicola* virulence.

In this study, subcellular localization assays showed that MgGPP can translocate from the ER to the nucleus. It has been suggested that PTM of effectors has an effect on their subcellular localization in plant cells [55]. For example, the cytoplasmically localized effector Hs10A07 is translocated to the nucleus after being phosphorylated [24]. Some studies also showed that proteolytic processing plays a role in the proper trafficking of effectors to their cellular targets. For example, the cyst nematode effector HsUbil was cleaved in plants, leading to the transport of the C-terminal domain into the nucleus [56]. Autoproteolysis of the *P*. *syringae* effector AvrPphB occurred inside plant cells to expose a myristoylation motif, and AvrPphB was then transported from the cytoplasm to the plasma membrane [32]. Interestingly, the C-terminal truncation mutation assays in our study showed that the C-terminal region of 123 aa to 224 aa of MgGPP was proteolytically cleaved *in planta*. Subcellular localization assays showed that eGFP:MgGPP^Δsp_Δ123–224^ was not located in the ER, WAK2ss:eGFP:MgGPP^Δsp_Δ123–224^:HDEL was always located in the ER, and WAK2ss:eGFP:MgGPP^Δsp^:HDEL was translocated from the ER to the nucleus. The C-terminal HDEL is a well-known signal for the retention of secretory proteins in the ER [57]; therefore, these observations demonstrated that the C-terminal region of 123 aa to 224 aa plays a role in the trafficking of MgGPP to the ER. MgGPP^Δsp_Δ123–224^ was exported from the ER due to the proteolytic processing of the C-terminal region (123 to 224) in the ER. Moreover, our data indicated that MgGPP^Δsp^ lacking the C-terminal region, i.e., MgGPP^Δsp_Δ123–224^, cannot be glycosylated, but the intact MgGPP^Δsp^ and WAK2ss:eGFP:MgGPP^Δsp_Δ123–224^:HDEL can be glycosylated. Glycosylated MgGPP can suppress the HR induced by Gpa2/RBP-1, but non-glycosylated MgGPP cannot. MgGPP^Δsp^ lacking the C-terminal region cannot suppress the host HR either. It has been reported that *N*-glycosylation of proteins in eukaryotic cells usually occurs in the ER [33]. These provide further evidence that the MgGPP^123-224^ region is required for MgGPP to translocate to the ER, and the effector MgGPP was glycosylated only when it was in the ER, after which the glycosylated MgGPP could be activated to suppress the HR.

In summary, we obtained a novel effector, MgGPP, from *M*. *graminicola*. Our experimental evidence suggests that MgGPP may be secreted into the host plants during parasitism, first into the cell apoplast, then entering into cells and targeted to the ER, where *N*-glycosylation and C-terminal proteolysis occurs, and it is finally translocated from the ER to the nucleus. *N*-glycosylated MgGPP suppressed host defenses and promoted the parasitism of *M*. *graminicola*. Our data provide an intriguing example of host-dependent proteolysis in concert with the glycosylation of a pathogen effector, suggesting that plant pathogen effectors can recruit the host post-translational machinery to mediate their PTM and regulate their activity to facilitate parasitism.

## Materials and methods

### Ethics statement

No specific permissions were required for the nematode collected for this study in Hainan Province, China. The field for nematodes collection was neither privately owned nor protected, and did not involve endangered or protected species.

### Nematodes and plant materials

*Meloidogyne graminicola* were collected from rice in Hainan, China, purified using a single egg mass, and reared on rice (*Oryza sativa* cv. ‘Nipponbare’) in a greenhouse at 28°C under 16:8 h light:dark conditions. Pre-J2 and parasitic stage nematodes were collected as described previously [29]. Rice (including wild-type and transgenic lines) and tobacco (*N*. *benthamiana*) were grown in a glasshouse at 28°C and 23°C, respectively, under 16:8 h light:dark conditions [29].

### Gene amplification and sequence analysis

Genomic DNA and total RNA were isolated from freshly hatched pre-J2s using the Genomic DNA Purification Kit (Shenergy Biocolor, Shanghai, China) and TRIzol reagent (Invitrogen, California, USA), respectively. Based on *M*. *graminicola* transcriptome data [29], the full-length cDNA sequence of *MgGPP* was obtained by rapid amplification of cDNA ends using a BD SMART RACE cDNA Amplification Kit (Clontech, California, USA) according to the manufacturer’s instructions. The genomic DNA was amplified using the primers MgGPP-gDNA-F/MgGPP-gDNA-R. All primers used in this study were synthesized by Invitrogen Biotechnology Co. Ltd. and are listed in S1 Table.

The sequence homology of the predicted proteins was analyzed using a BLASTx, BLASTn or tBLASTn search of the nonredundant and Expressed Sequence Tags database of the National Center for Biotechnology Information (http://www.ncbi.nlm.nih.gov/BLAST/). The signal peptide was predicted using SignalP 4.0 (http://www.cbs.dtu.dk/services/SignalP/). Putative transmembrane domains were predicted based on TMHMM (http://www.cbs.dtu.dk/services/TMHMM/). Molecular mass was predicted using ProtParam, and motif analyses were performed using InterProScan [58]. Glycosylation was analyzed using NetNGlyc1.0 (http://www.hiv.lanl.gov/content/sequence/GLYCOSITE/glycosite.html). Nuclear localization signal (NLS) domains were predicted as previously described [59].

### Plasmid construction and generation of transgenic rice plants

First, the CaMV35S-promotor of the pCAMBIA1305.1 vector was replaced with the maize ubiquitin promoter to generate the binary vector pUbi (S7A Fig). Subsequently, for overexpression constructs, the coding sequence of MgGPP^Δsp^ was cloned into the pUbi vector (S7B Fig). For *MgGPP* silencing constructs, the fragment of 251 to 469 bp within the *MgGPP* sequence was selected as the RNAi target and confirmed to have no contiguous 21-nucleotide identical hit in other genes. *MgGPP*^*251-469*^ was inserted into the pMD-18T vector (Takara, Tokyo, Japan) in both sense and antisense orientations. The sense and antisense fragments were separated by a GUS intron. Then, the entire RNAi fragment was inserted into the pUbi vector to generate transgenic rice lines expressing hairpin dsRNA (S7C Fig). The overexpression and RNAi constructs were transformed into *A*. *tumefaciens* strain EHA105 and used to transform the rice calli. The transgenic seedlings were screened on N6 Medium containing 50 mg/L hygromycin [60].

The expression levels of *MgGPP* in each transgenic rice line were determined by qRT-PCR. RNAi transgenic lines were confirmed using the gus intron fragment as target and the *MgGPP* expressions were determined in nematodes extracted from roots of RNAi lines and the control lines. The *OsUBQ* and *Mg-ACT2* genes were selected as the reference gene for qRT-PCR, and two other genes, *Mg-CRT* and *Mg-expansin*, were used to verify the specificity of *MgGPP*-targeting RNAi by qRT -PCR analysis. Three technical replicates of each reaction were performed in all experiments and three independent experiments were performed. Expression levels of the transgenic lines were calculated using the 2^-ΔΔCT^ method. Western blot analysis was performed to determine MgGPP expression in transgenic lines. Total proteins were extracted from each transgenic lines, control lines and *Meloidogyne graminicola*.

### Southern blot analysis

Ten micrograms of *M*. *graminicola* total genomic DNA were separately digested with *Hind*III (no cleavage site within *MgGPP*) or *Sph*I (one cleavage site located at 389 to 394 bp) before separation by electrophoresis and then transferred to Hybond N^+^ membranes (Amersham Biosciences, Buckinghamshire, UK). The probe hybridization and signal detection were performed as previously described [10]. The digoxigenin-labeled DNA probe targeting the region from 91 bp to 366 bp of the *MgGPP* gene was synthesized using a PCR DIG probe synthesis kit (Roche Applied Science, Rotkreuz, Switzerland).

### Developmental expression analysis and *in situ* hybridization

RNA samples were prepared from approximately 100 *M*. *graminicola* nematodes at different life stages as indicated using the RNA prepmicro kit (Tiangen Biotech, Beijing, China). The cDNA was synthesized using TransScript All-in-One First-Strand cDNA Synthesis SuperMix (Transgen Biotech, Beijing, China). qRT-PCR was performed using the primer pairs Mg-qPCR-F/Mg-qPCR-R and Mg*-*ACT2-F/Mg*-*ACT2-F-R for amplifying the *MgGPP* gene and the endogenous reference gene *Mg-ACT2*, respectively [29]. qRT-PCR was performed using the SYBR Premix Ex Taq II (Tli RNaseH Plus) (Takara, Tokyo, Japan) on a Thermal Cycler Dice Real Time System (Takara, Tokyo, Japan). Three technical replicates for each reaction were performed in all experiments, and three independent experiments were performed. The relative changes in gene expression were determined using the 2^-ΔΔCT^ method [61].

For *in situ* hybridization, approximately 10,000 freshly hatched *M*. *graminicola* pre-J2s were collected as described previously [29]. Digoxigenin (DIG)-labeled probes were synthesized as described above. The nematode sections were hybridized as described previously [41] and examined by microscopy using a Nikon ECLIPSE Ni microscope (Nikon, Tokyo, Japan). Three independent experiments were performed.

### Anti-MgGPP polyclonal serum production and immunofluorescence localization

The anti-MgGPP polyclonal serum was obtained as described previously [39]. Briefly, the MgGPP protein was expressed in BL21 (DE3) cells and purified using Ni^2+^NTA agarose (Merck) according to the user manual. The amount and the purity of the purified protein were determined by the BCA method (Tiangen Biotech, Beijing, China) and sodium dodecyl sulfate–polyacrylamide gel electrophoresis (SDS-PAGE). The anti-MgGPP polyclonal serum was obtained by immunizing rabbits.

For immunolocalization on sections of rice galls, rice galls infected with *M*. *graminicola* for 5 days were dissected, fixed, dehydrated and embedded in paraffin as described previously [42]. Sections were incubated in dimethylbenzene and an alcohol gradient to remove the paraffin. Then, the sections were treated with an MgGPP primary antibody at room temperature for 2 h in a humid box. They were washed three times for 5 min using PBS and then incubated with Goat anti-Rabbit Superclonal Secondary Antibody, Alexa Fluor 488 conjugate (Thermo Fisher Scientific, San Jose, CA, USA) at room temperature for 2 h in a humid box. Finally, the sections were mounted with Fluoromount-G (SouthernBiotech, Birmingham, UK) containing DAPI and observed with a Nikon ECLIPSE Ni microscope.

### Subcellular localization

For constructing the MgGPP^Δsp^:eGFP, eGFP:MgGPP^Δsp^ and eGFP plasmids, the sequences of MgGPP^Δsp^ and eGFP were amplified and cloned into pUbi. As a control, mCherry was cloned into WAK2ss:HDEL to generate WAK2ss:mCherry:HDEL. The protoplasts of rice root tissues were obtained as previously described [62]. Then, 50 μg of the MgGPP^Δsp^:eGFP, eGFP:MgGPP^Δsp^ and eGFP plasmids were added to 1 mL (approximately 2 × 10^3^ cells) of rice protoplasts, respectively. The protoplasts were then incubated in the dark at room temperature for ~8 and ~48 h and examined under a Nikon ECLIPSE Ni microscope.

### Western blot analysis

For verification of the intact fusion protein, western blot analysis was performed as described previously [9]. Briefly, total proteins from rice root protoplasts and tobacco leaves were separately extracted using RIPA lysis buffer (2% SDS, 80 mM Tris/HCl, pH 6.8, 10% glycerol, 0.002% bromophenol blue, 5% β-mercaptoethanol and Complete protease inhibitor cocktail). Approximately 20 μg of total proteins were separated on a 12% SDS-polyacrylamide gel electrophoresis (SDS-PAGE) gel and transferred to a nitrocellulose membrane (PALL, Washington, NY, USA). The membranes were blocked with 5% (w/v) nonfat dry milk, incubated with a primary mouse anti-GFP antibody (Transgene Biotech, Beijing, China) at a 1:3000 dilution, and then incubated with an anti-mouse horseradish peroxidase-conjugated secondary antibody at a 1:2000 dilution (Biosynthesis Biotechnology Co., Beijing, China). The proteins were visualized using the Immobilon Western Chemiluminescent system with Pierce ECL Western Blotting Substrate (Thermo Fisher Scientific, San Jose, CA, USA).

### *N*-Glycosylation analysis

For glycosylation analysis, the cDNA of MgGPP^Δsp^ was amplified and cloned into the pCAMBIA1305.1 and pUbi vectors. The two constructs, pCAMBIA:eGFP:MgGPP^Δsp^ and eGFP:MgGPP^Δsp^, were expressed in tobacco leaves and rice root protoplasts, respectively. Total protein extracted with RIPA lysis buffer was digested with PNGase A (New England Biolabs, Beverly, MA, USA) for 1 h before being mixed with the loading buffer. Meanwhile, the total protein samples from pre-J2s, para-J2s/J3s and females of *M*. *graminicola* were treated with or without PNGase F (New England Biolabs, Beverly, MA, USA). PNGase F and PNGase A were used for glycosylation analysis of MgGPP following the manufacturer’s instructions. The extracted protein samples from three independent transformations were analyzed by western blot.

For glycosylation site analysis, MgGPP^Δsp^ was used to mutate the putative *N*-glycosylation site at Asn-110 to Gln by a PCR-mediated approach. Subsequently, the mutant MgGPP^Δsp-N110Q^ was cloned into the pCAMBIA1305.1 and pUbi vectors. Finally, the constructs pCAMBIA:eGFP:MgGPP^Δsp-N110Q^ and eGFP:MgGPP^Δsp-N110Q^ were obtained and expressed in tobacco leaves and rice root protoplasts, respectively. The extracted protein samples from three independent transformations were analyzed by western blot.

### Proteolysis analysis

Different cDNAs of MgGPP mutants as indicated were amplified and cloned into the pUbi vector. In addition, MgGPP^Δsp^ and MgGPP^Δsp_Δ123–224^ were cloned into WAK2ss:HDEL to generate the WAK2ss:eGFP:MgGPP^Δsp^:HDEL and WAK2ss:eGFP:MgGPP^Δsp_Δ123–224^:HDEL constructs. All of the splicing- and mutagenesis-generated MgGPP mutants in this work were obtained by PCR-driven overlap extension [63]. These mutant constructs were expressed in rice root protoplasts and examined under a Nikon ECLIPSE Ni microscope. The extracted protein samples from three independent transformations were analyzed by western blot.

### Infection assay

Twelve-day-old rice plants were inoculated with 200 *M*. *graminicola* pre-J2s. At 12 dpi, the roots were collected, washed and stained by acid fuchsin, and the number of females was counted. Each experiment was performed three times. Statistically significant differences between treatments were determined by an unadjusted paired t-test (P<0.05) with SAS version 9.2 (SAS Institute, North Carolina, USA).

### Cell death assay

*N*. *benthamiana* plants were grown at 23°C for 4 weeks in 16 h light: 8 h dark conditions in a greenhouse. All mutant sequences of MgGPP were cloned into the pCAMBIA1305.1 vector to generate the pCAMBIA:flag:MgGPP^Δsp^, pCAMBIA:flag:MgGPP^Δsp_Δ123–224^ and pCAMBIA:flag:MgGPP^Δsp-N110Q^ fusion constructs. The Gpa2, RBP–1, INF1 and GrCEP12 sequences were synthesized by Generay Biotech Co., Ltd. and cloned into pCAMBIA1305.1 to generate the expression constructs pCAMBIA1305:GrCEP12, pCAMBIA1305:Gpa2, pCAMBIA1305:INF1:HA and pCAMBIA1305:RBP-1:HA. The other elicitor of programmed cell death, the pCAMBIA1305:Bax construct, was generated as described previously [9]. All constructs were introduced into *A*. *tumefaciens* GV3101, and the transformed bacteria were then suspended in a buffer containing 10 mM 2-(N-morpholino) ethanesulfonic acid (MES) (pH 5.5) and 200 μM acetosyringone at an absorbance at 600 nm (OD600) of 0.3. *A*. *tumefaciens* cells carrying the constructs pCAMBIA:flag:MgGPP^Δsp^, pCAMBIA:flag:MgGPP^Δsp_Δ123–224^ and pCAMBIA:flag:MgGPP^Δsp-N110Q^ were infiltrated into *N*. *benthamiana* leaves as described previously [9,12]. After 24 h, the same infiltration sites were injected with *A*. *tumefaciens* cells carrying the constructs pCAMBIA1305:Gpa2/pCAMBIA1305:RBP-1:HA, pCAMBIA1305:Bax and pCAMBIA1305:INF1:HA. As controls, the buffer and *A*. *tumefaciens* carrying the empty vector pCAMBIA1305 and pCAMBIA1305:GrCEP12 were separately infiltrated in parallel. Photographs were taken for symptom analysis 5 days after the last infiltration. The cell-death phenotype was scored by a necrosis index [64].

To confirm gene expression, western blotting and RT-PCR were performed. Western blot analysis was performed as described above. RT-PCRs were performed using the gene-specific primers MgGPP-F/MgGPP-R, Bax-F/Bax-R, RBP-1-F/RBP-1-R, Gpa2-F/Gpa2-R, INF1-F/INF1-R and *NbEF1*α-F/*NbEF1*α-R to amplify MgGPP, Gpa2, RBP-1, INF1 and the control *NbEF1*α [65], respectively.

## Supporting information

S1 Table Primers used in this study. (PDF)

S1 Fig Sequence analysis of *MgGPP*. (A) The DNA sequence of *MgGPP*. The predicted start codon and stop codon are in red; the two introns are presented in italic and lower-case letters; and the untranslated regions are bold. (B) Putative amino acid sequence of MgGPP. The predicted signal peptide is underlined; a putative SV40-like NLS domain is boxed; and a predicted *N*-glycosylation site is in red.
(TIF)

S2 Fig Southern blot analysis of *MgGPP*. *MgGPP* is a single copy gene in the *Meloidogyne graminicola* genome. Genomic DNA of *M*. *graminicola* was digested with *HindIII* and *SphI* and probed with a digoxigenin-labeled 300-bp fragment of *MgGPP* DNA.
(TIF)

S3 Fig Purification of recombinant MgGPP and anti-MgGPP serum specifically reacts with MgGPP. (A) Purification of recombinant pET32a-MgGPP. SDS-PAGE (12%) analysis of the recombinant MgGPP protein (red box) stained with Coomassie brilliant blue; 1–2, binding buffer; 3–5, wash buffer; 6, elute buffer. M, the protein standard molecular weight. (B) Western blot analysis of total proteins (10 μg) from pre-J2s and healthy rice roots (RIT) with pre-immune serum (left) or anti-MgGPP serum (right).
(TIF)

S4 Fig Immunodetection of the MgGPP protein in sectioned rice galls. (A-D) Galls containing a nematode at 5 days postinfection (dpi) incubated with pre-immune serum, showing no signal. (E-H) Galls containing a nematode at 5 dpi without any treatment, showing no signal. (I-L) Healthy rice roots incubated with anti-MgGPP serum, showing no signal. Micrographs A, E and I are observations of the Alexa Fluor 488-conjugated secondary antibody. Micrographs B, F and J are images of 4,6-diamidino-2-phenylindole (DAPI)-stained nuclei. Micrographs C, G and K are images of differential interference contrast. Micrographs D, H and L are superpositions of images of the Alexa Fluor 488-conjugated secondary antibody, DAPI-stained nuclei and differential interference contrast. N, nematode; H, the head of nematode; asterisks, giant cells; Scale bars, 20 μm.
(TIF)

S5 Fig Southern blot analysis of transgenic rice lines and RT-PCR confirmation of transgenic lines. (A) and (C) Total gDNA was extracted from rice roots of overexpression and RNAi lines and wild-type (WT) controls. The genomic DNA was digested with the restriction endonuclease *Hind*III and then hybridized on blots with an *MgGPP* digoxigenin (DIC)-labeled probe, showing single-copy transgenic lines (red arrows). (B) and (D) RT-PCR was used to confirm the expression of MgGPP and the GUS intron in transgenic overexpression lines and RNAi lines compared with the WT control. OE-4, 5, 6, 9 and 39, five transgenic rice lines expressing *MgGPP*; P, positive control; WT, wild type. RNAi 6, 15, 25 and 26, different transgenic RNAi rice lines. M, standard molecular weight.
(TIF)

S6 Fig Suppression of Bax- and INF1-triggered cell death by MgGPP. (A) Assay of the suppression of Bax- and INF1**-**triggered cell death in *Nicotiana benthamiana* by MgGPP. *N*. *benthamiana* leaves were infiltrated with buffer or *Agrobacterium tumefaciens* cells carrying MgGPP^Δsp^, MgGPP^Δsp_Δ123–224^, MgGPP^Δsp_N110Q^ and the flag control gene alone or followed 24 h later with *A*. *tumefaciens* cells carrying the Bax or INF1 genes. The cell death phenotype was scored, and photographs were taken 5 days after the last infiltration. (B) The average areas of cell death of in leaves infiltrated with cells carrying MgGPP and other proteins followed by Bax or INF1. Statistical significance of the necrosis index of MgGPP and other proteins compared with that of the negative control flag. Each column represents the mean with standard deviation (n = 55). *P<0.05, **P<0.01, Student’s t test.
(TIF)

S7 Fig The scheme of the constructs used in rice transformation. (A) The CaMV35S-promotor of pCAMBIA1305.1 vector was replaced with the maize ubiquitin promoter to generate the binary vector pUbi. (B) Schematic of the full-length *MgGPP* construct. (C) Constructs generated for *MgGPP* overexpression (OE) and (D) host-induced RNA interference (RNAi).
(TIF)
