# Supplementary material for: A novel Meloidogyne graminicola effector, MgGPP, is secreted into host cells and undergoes glycosylation in concert with proteolysis to suppress plant defenses and promote parasitism
Source: PLoS Pathog. 2017 Apr 12;13(4):e1006301. doi: 10.1371/journal.ppat.1006301 (PMC5402989; doi:10.1371/journal.ppat.1006301)
Supplement: S1 Table — (PDF) [file ppat.1006301.s001.pdf]

S1 Table. Primers used in this study.

Primer	Sequence 5'- 3'	Purpose
MgGPP-race-F	CAATTCCAGTTATGGATGAAAC	*MgGPP* gene 3' flanking region PCR amplification
MgGPP-race-R	AAGGCTCCACCAACAGCAGCA	*MgGPP* gene 5' flanking region PCR amplification
MgGPP-gDNA-F	TAGTGAGTCGTATTACGGCC	*MgGPP* genomic fragment PCR amplification
MgGPP-gDNA-R	GTTCTTCTTTATATCTTCGAT	
MgGPP-ORF-F	ATGACAAAAAATACTTCTTAC	*MgGPP* open reading frame region PCR amplification
MgGPP-ORF-R	TTATTTTGGACATTTTAGAT	
Southern-F	TGTCCAGAAATACCCTCAAATGGT	Southern blot probe amplification in *M. graminicola* or transgenic lines
Southern-R	ACCACATTCACCACCATAAGCA	
In-situ-HYBRID-F	ACCAGTGCATAATTGTCCAGA	*In situ* hybridization assays in *M. graminicola*
In-situ-HYBRID-R	CGGAAAAGAAGGCTCCACCA	
MgGPP-qPCR-F	TTACGCCACAAGAGACTG	qRT-PCR for *MgGPP* expression patterns in *M. graminicola*
MgGPP-qPCR-R	CACATTCACCACCATAAGC	
GUS-qPCR-F	TTCTTGGTTAGGACCCTTT	qRT-PCR for *gus* intron expression in RNAi lines
GUS-qPCR-R	AGTTCGTCGGTTCTGTAA	
GUS-F	AAGCTTGTAGATCTGAGGGTAAA	GUS intron region PCR amplification
GUS-R	CTGCAGCTAGTTCGTCGGTTCTGTAA	
Mg-CRT-F	TTCTTCTCTTCCTCTTCA	qRT-PCR for *MgCRT* expression in *M. graminicola* affecting RNAi lines
Mg-CRT-R	GTGCTGTTGGTATTGATA	
Mg-EXPANSION-F	CTCAGGCTCTTTATTTACATCA	qRT-PCR for *Mg-expansion* expression in *M. graminicola* affecting RNAi lines
Mg-EXPANSION-R	TTCGCATTCAGGACATTG	
Mg-ACT2-F	ATGGCAACTGCCGCTTCTTCT	*M. graminicola* housekeeping gene (*ACT2*) used as qRT-PCR reference
Mg-ACT2-R	AGATTCCGGACAACGGAAGCGT	
pET32a-MgGPP-F	GGATCCATGGAAATCAAAAAATATAAAC	pET32a:MgGPP constructed for prokaryotic expression
pET32a-MgGPP-R	GAATTCTTACTTGTACAGCTCGTCCA	
NbEF1α-F	AAGGTCCAGTATGCCTGGGTGCTTGAC	*Nicotiana benthamiana* housekeeping gene (*NbEF1α*) amplification, used as qRT-PCR reference
NbEF1α-R	AAGAATTCACAGGGACAGTTCCAATACCA	
MgGPP-BamHI-F	GGATCCATGGAAATCAAAAAATATAAAC	MgGPP$^{\Delta sp}$:eGFP constructed for transient expression assays in rice protoplasts
MgGPP-PstI-R	CTGCAGCTTGTACAGCTCGTCCA	
eGFP-PstI-F	CTGCAGGTGAGCAAGGGCGAGGAGCT	
eGFP-MluI-R	ACGCGTTTACTTGTACAGCTCGTCCA	
MgGPP–PstI-F	CTGCAGGAAATCAAAAAATATAAAC	eGFP:MgGPP$^{\Delta sp}$ constructed for transient expression assays in rice protoplasts
MgGPP –MluI-R	ACGCGTTTATTTTGGACATTTTAGAT	
eGFP-BamHI-F	GGATCCATGGTGAGCAAGGGCGAGGAGCT	
eGFP-PstI-R	CTGCAGTTACTTGTACAGCTCGTCCA	
Ubi-eGFP-BamHI-F	CTGCAGATGGTGAGCAAGGGCGAGGAGCT	Ubi:eGFP constructed for transient expression assays in rice protoplasts
Ubi-eGFP-MluI-R	ACGCGTTTACTTGTACAGCTCGTCCA	
Ubi-mCherry-BamHI-F	GGATCCATGGTGAGCAAGGGCGAGGA	Ubi:mCherry constructed for transient expression assays in rice protoplasts
Ubi-mCherry-MluI-R	ACGCGTTTACTTGTACAGCTCGTCCAT	
WAKA2ss-F	ATGAAGGTACAGGAGGGTTT	WAKA2ss amplification
WAKA2ss-R	CCTCCTCGCCCTTGCTCACCATGGATCCGCACTCCTTGCG	

WAKA2ss-mCherry-HDEL-F	GTTGTTAGGTGTTACTTCTGAAGAG ATGAAGGTACAGGAGGGTTTGTTC	WAKA2ss:mCherry:HDEL constructed for transient expression assays in rice protoplasts
WAKA2ss-mCherry-HDEL-R	TGGTCACCAATTCACAAAAACGCGTTTACAGCTCGTCAT GAGATCTACGCGT	
MgGPP^{sp}-eGFP-HDEL-F	GGATCCTTTTGGACATTTTAGATAAG	WAKA2ss:MgGPP$^{\Delta\ sp}$:eGFP:HDEL constructed for transient expression assays in rice protoplasts
MgGPP$^{\Delta\ sp_\Delta 123-224}$-eGFP-HDEL-R	GGATCCACCACATTCACCACCATAAG	WAKA2ss:MgGPP$^{\Delta sp_\Delta 123-224}$:eGFP:HDEL constructed for transient expression assays in rice protoplasts
MgGPP$^{\Delta sp-N110Q}$-F	ATTTTTTATTTTCATGCGTATCAGGACAGTGATAAACATG CTTA	eGFP:MgGPP$^{\Delta sp_N110Q}$ constructed for transient expression assays in rice protoplasts and *N. benthamiana*
MgGPP$^{\Delta sp-N110Q}$ -R	CTGATACGCATGAAAATAAAAAATTC	
MgGPP$^{\Delta sp_\Delta 121-224}$-eGFP-R	CTGCAGTTCACCACCATAAGCATGTTTAT	MgGPP$^{\Delta sp_\Delta 121-224}$-eGFP constructed for transient expression assays in rice protoplasts
MgGPP$^{\Delta sp_\Delta 141-224}$-eGFP-R	CTGCAGACCGGAAAAGAAGGCTCCACCAAC	MgGPP$^{\Delta sp_\Delta 141-224}$-eGFP constructed for transient expression assays in rice protoplasts
MgGPP$^{\Delta sp_\Delta 161-224}$-eGFP-R	CTGCAG ATCATGTATAGAATGATATGAA	MgGPP$^{\Delta sp_\Delta 161-224}$-eGFP constructed for transient expression assays in rice protoplasts
MgGPP$^{\Delta sp-200-224}$-eGFP-R	CTGCAG TGCCTCCTCAGATAAATCAAGT	MgGPP$^{\Delta sp_\Delta 200-224}$:eGFP constructed for transient expression assays in rice protoplasts
MgGPP$^{\Delta sp_\Delta 122-224}$-eGFP-R	CTGCAGACATTCACCACCATAAGCATGTTTAT	MgGPP$^{\Delta sp_\Delta 122-224}$:eGFP constructed for transient expression assays in rice protoplasts
MgGPP$^{\Delta sp_\Delta 123-224}$-eGFP-R	CTGCAG ACCACATTCACCACCATAAGCAT	MgGPP$^{\Delta sp_\Delta 123-224}$:eGFP constructed for transient expression assays in rice protoplasts
MgGPP$^{\Delta sp_\Delta 124-224}$-eGFP-R	CTGCAG TAGACCACATTCACCACCATAAG	MgGPP$^{\Delta sp_\Delta 124-224}$:eGFP constructed for transient expression assays in rice protoplasts
MgGPP$^{\Delta sp_\Delta 125-224}$-eGFP-R	CTGCAG TCCTAGACCACATTCACCACCAT	MgGPP$^{\Delta sp_\Delta 125-224}$:eGFP constructed for transient expression assays in rice protoplasts
MgGPP$^{\Delta sp_\Delta 126-224}$-eGFP-R	CTGCAGTACTCCTAGACCACATTCACCAC	MgGPP$^{\Delta sp_\Delta 126-224}$:eGFP constructed for transient expression assays in rice protoplast
MgGPP$^{\Delta sp_\Delta 121-140}$-eGFP-F	GCAACAAAAAAATTGGCCCCG	MgGPP$^{\Delta sp_\Delta 121-140}$:eGFP constructed for transient expression assays in rice protoplasts
MgGPP$^{\Delta sp_\Delta 121-140}$-eGFP-R	CGGGGCCAATTTTTTTGTTGCTTCACCACCATAAGCATG	MgGPP$^{\Delta sp_\Delta 121-140}$:eGFP constructed for transient expression assays in rice protoplasts
MgGPP$^{\Delta sp_\Delta 121-160}$-eGFP-F	GAAAAGGTTTGCATAGATAT	MgGPP$^{\Delta sp_\Delta 121-160}$:eGFP constructed for transient expression assays in rice protoplasts
MgGPP$^{\Delta sp_\Delta 121-160}$-eGFP-R	ATATCTATGCAAACCTTTTCTTCACCACCATAAGCATG	MgGPP$^{\Delta sp_\Delta 121-160}$:eGFP constructed for transient expression assays in rice protoplasts
MgGPP$^{\Delta sp_\Delta 121-200}$-eGFP-F	TTTAATTGGTTAAAACCTGA	MgGPP$^{\Delta sp_\Delta 121-200}$:eGFP constructed for transient expression assays in rice protoplasts
MgGPP$^{\Delta sp_\Delta 121-200}$-eGFP-R	TCAGGTTTTAACCAATTAAATTCACCACCATAAGCATG	MgGPP$^{\Delta sp_\Delta 121-200}$:eGFP constructed for transient expression assays in rice protoplasts
eGFP-MgGPP$^{\Delta sp_\Delta 123-224}$-R	ACGCGTTTATTCACCACCATAAGCATGTTTAT	eGFP:MgGPP$^{\Delta sp_\Delta 123-224}$ constructed for transient expression assays in rice protoplasts

pCAMBIA1305-GrCLE12-F	AAGCTTGGCACTGGCCGTCGT	pCAMBIA1305:GrCLE12 constructed for cell death assays in *N. benthamiana*
pCAMBIA1305-GrCLE12-R	CACGTGTTATCAGTGGTTCTCGCACTCGCAGGGTCCATGTCCACCAATCATGGTCAAGAGTCCCCCG	
pCAMBIA1305-MgGPP-F	CCATGGCCGATTACAAGGATGACGACGATAAGTGAGTTTAAACGAAATCAAAAAATATAAAC	pCAMBIA1305:flag:MgGPP$^{\Delta sp}$ and pCAMBIA1305-MgGPP$^{\Delta sp\text{-}N110Q}$-R constructed for cell death assays in *N. benthamiana*
pCAMBIA1305-MgGPP-R	CACGTGTTATTTTGGACATTTTAGAT	
pCAMBIA1305-MgGPP$^{\Delta sp_\Delta 123\text{-}224}$-R	CACGTGTTAACCACATTCACCACCATAAGCAT	pCAMBIA1305:flag:MgGPP$^{\Delta sp_\Delta 123\text{-}224}$ constructed for cell death assays in *N. benthamiana*
pCAMBIA1305-Bax-F	CCATGGATGGACGGGTCCGGGGAGC	pCAMBIA1305:Bax constructed for cell death assays in *N. benthamiana*
pCAMBIA1305-Bax-R	CACGTGTTAGCCCATCTTCTTCCAGATGG	
pCAMBIA1305-RBP-1-F	GAACACGGGGGACTCTTGACCATGATGCGCACCTTTCTCTTCTC	pCAMBIA1305:RBP-1:HA constructed for cell death assays in *N. benthamiana*
pCAMBIA1305- RBP-1-R	TCGAGCTGGTCACCAATTCACACGTGAGCGTAATCTGGAACATCGTATGGGTATAAATTCTCGTTTTTCAGTTTC	
pCAMBIA1305-Gpa2-F	GAACACGGGGGACTCTTGACCATGGCTTATGCTGCTGTTAC	pCAMBIA1305-Gpa2 constructed for cell death assays in *N. benthamiana*
pCAMBIA1305-Gpa2-R	CGAGCTGGTCACCAATTCACACGTGTCACACTACCAAAAATGCTCCAT	
pCAMBIA1305-INF1-F	GAACACGGGGGACTCTTGACCATGAACTTTCGTGCTCTGTTCG	pCAMBIA1305:INF1:HA constructed for cell death assays in *N. benthamiana*
pCAMBIA1305-INF1-R	TCGAGCTGGTCACCAATTCACACGTGAGCGTAATCTGGAACATCGTATGGGTACACGTGTAGCGATGCACACGTAGAC	
MgGPP-F	TGTCCAGAAATACCCTCAAAT	*MgGPP* gene amplified in cell death assays in *N. benthamiana*
MgGPP-R	CAGTCTCTTGTGGCGTAAA	
OsUBQ-F	CCAGTAAGTCCTCAGCCATGGAG	Rice housekeeping gene (*OsUBQ*, Os03g13170) used as qRT-PCR reference
OsUBQ-R	GGACACAATGATTAGGGATC	

Restriction enzyme sequences are in red font.
