# Supplementary figures and images for: A novel Meloidogyne graminicola effector, MgGPP, is secreted into host cells and undergoes glycosylation in concert with proteolysis to suppress plant defenses and promote parasitism
Source: PLoS Pathog. 2017 Apr 12;13(4):e1006301. doi: 10.1371/journal.ppat.1006301 (PMC5402989; doi:10.1371/journal.ppat.1006301)

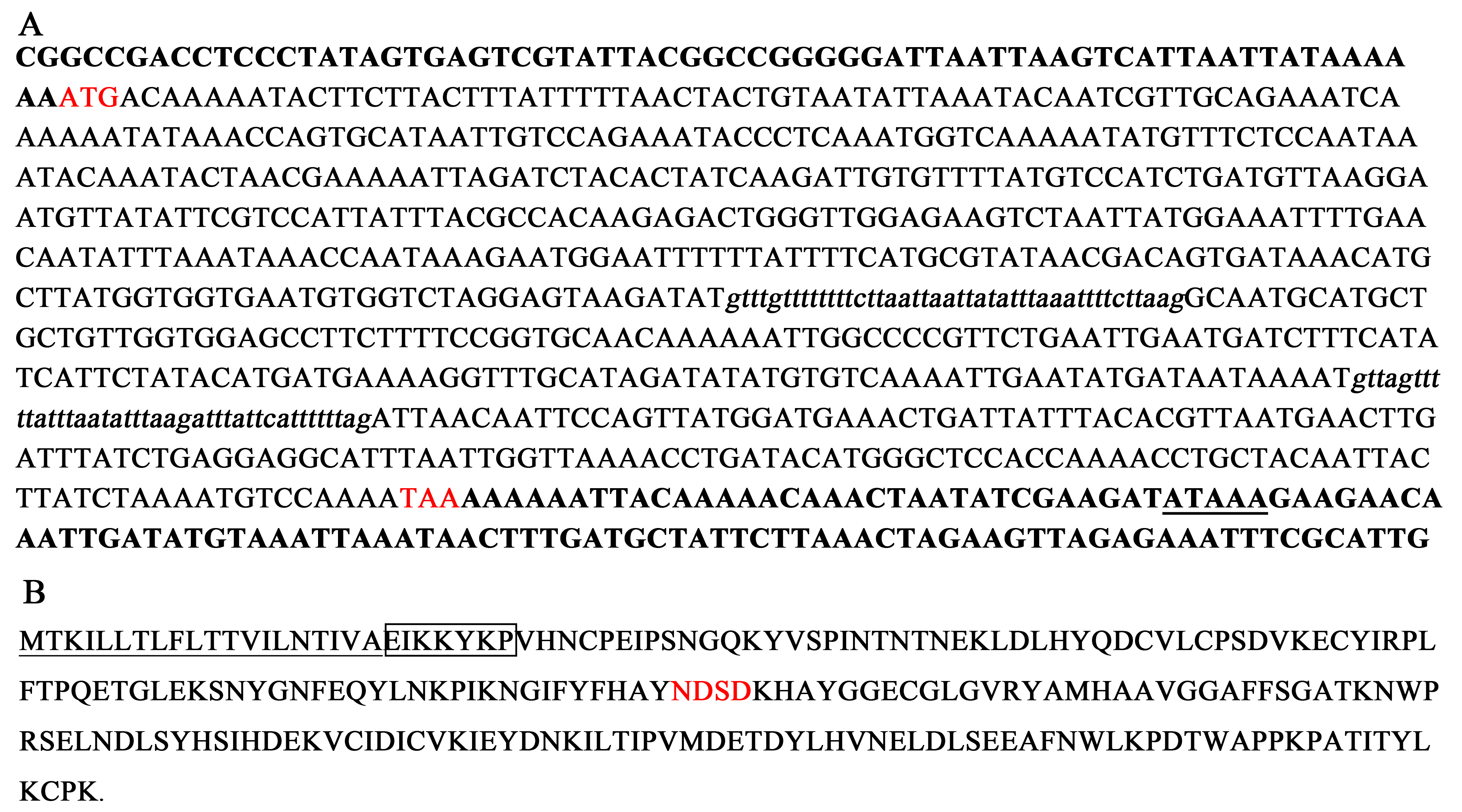

Supplement: S1 Fig — (A) The DNA sequence of MgGPP. The predicted start codon and stop codon are in red; the two introns are presented in italic and lower-case letters; and the untranslated regions are bold. (B) Putative amino acid sequence of MgGPP. The predicted signal peptide is underlined; a putative SV40-like NLS domain is boxed; and a predicted N-glycosylation site is in red. (TIF) [file ppat.1006301.s002.tif]

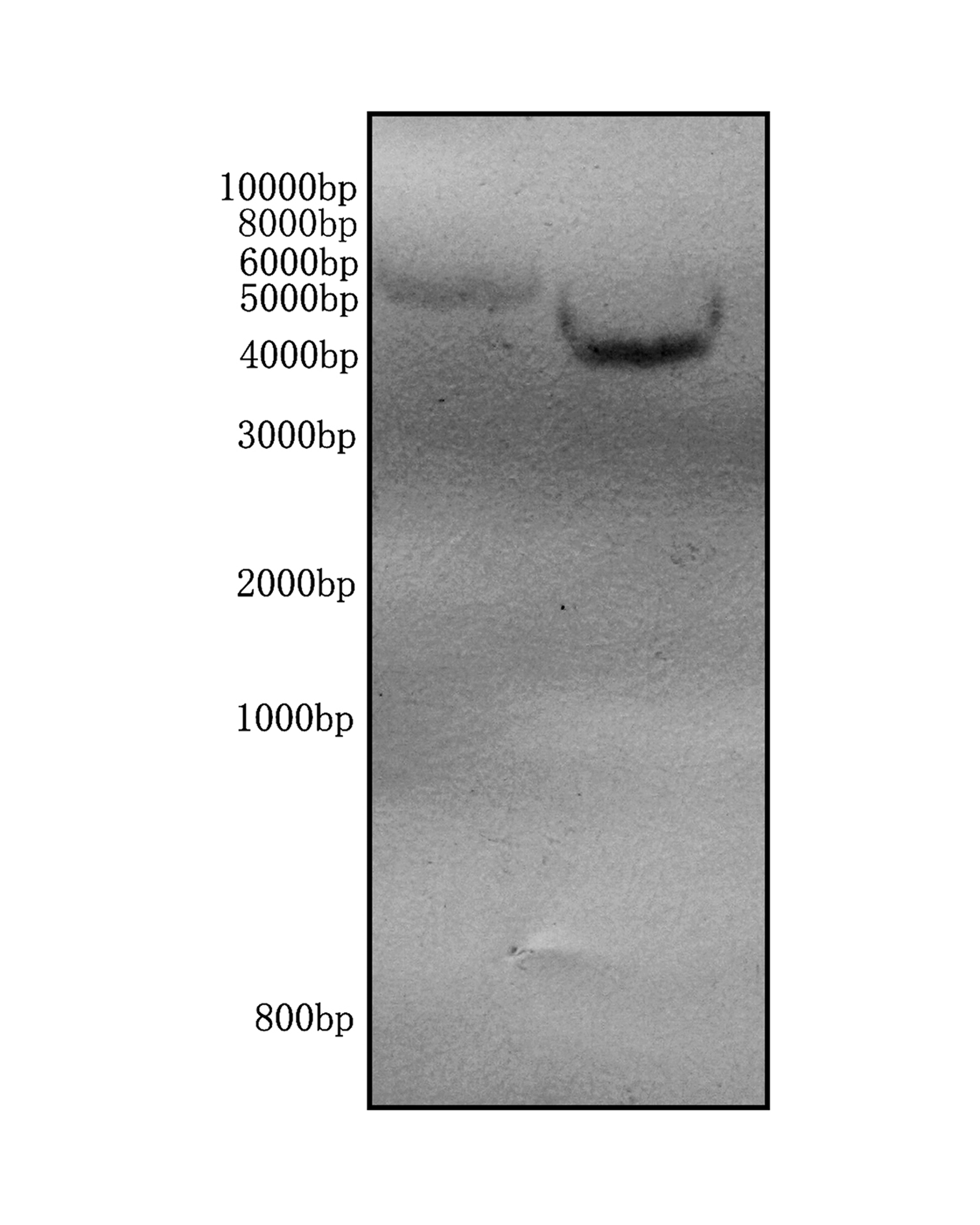

Supplement: S2 Fig — MgGPP is a single copy gene in the Meloidogyne graminicola genome. Genomic DNA of M. graminicola was digested with HindIII and SphI and probed with a digoxigenin-labeled 300-bp fragment of MgGPP DNA. (TIF) [file ppat.1006301.s003.tif]

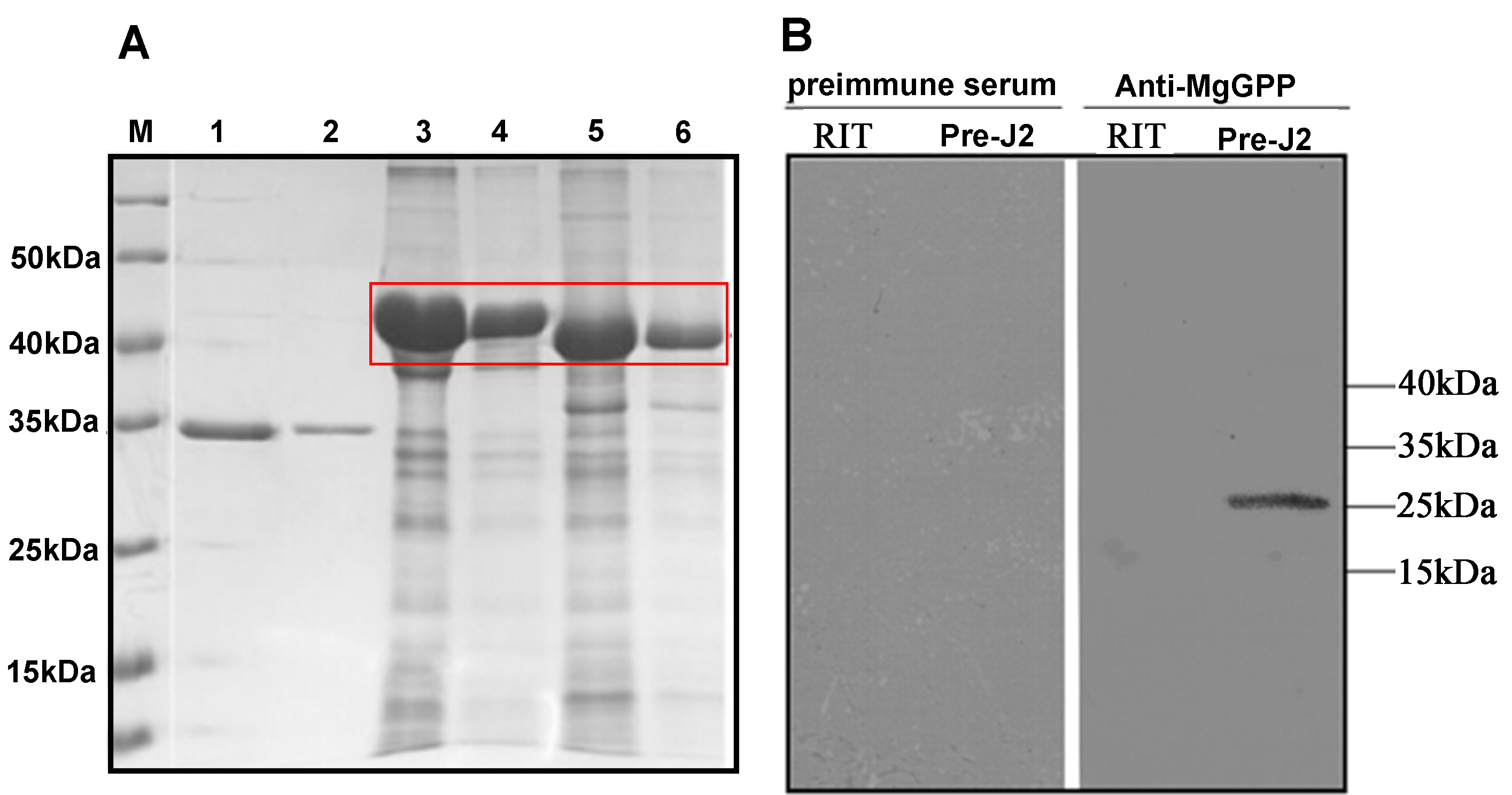

Supplement: S3 Fig — (A) Purification of recombinant pET32a-MgGPP. SDS-PAGE (12%) analysis of the recombinant MgGPP protein (red box) stained with Coomassie brilliant blue; 1–2, binding buffer; 3–5, wash buffer; 6, elute buffer. M, the protein standard molecular weight. (B) Western blot analysis of total proteins (10 μg) from pre-J2s and healthy rice roots (RIT) with pre-immune serum (left) or anti-MgGPP serum (right). (TIF) [file ppat.1006301.s004.tif]

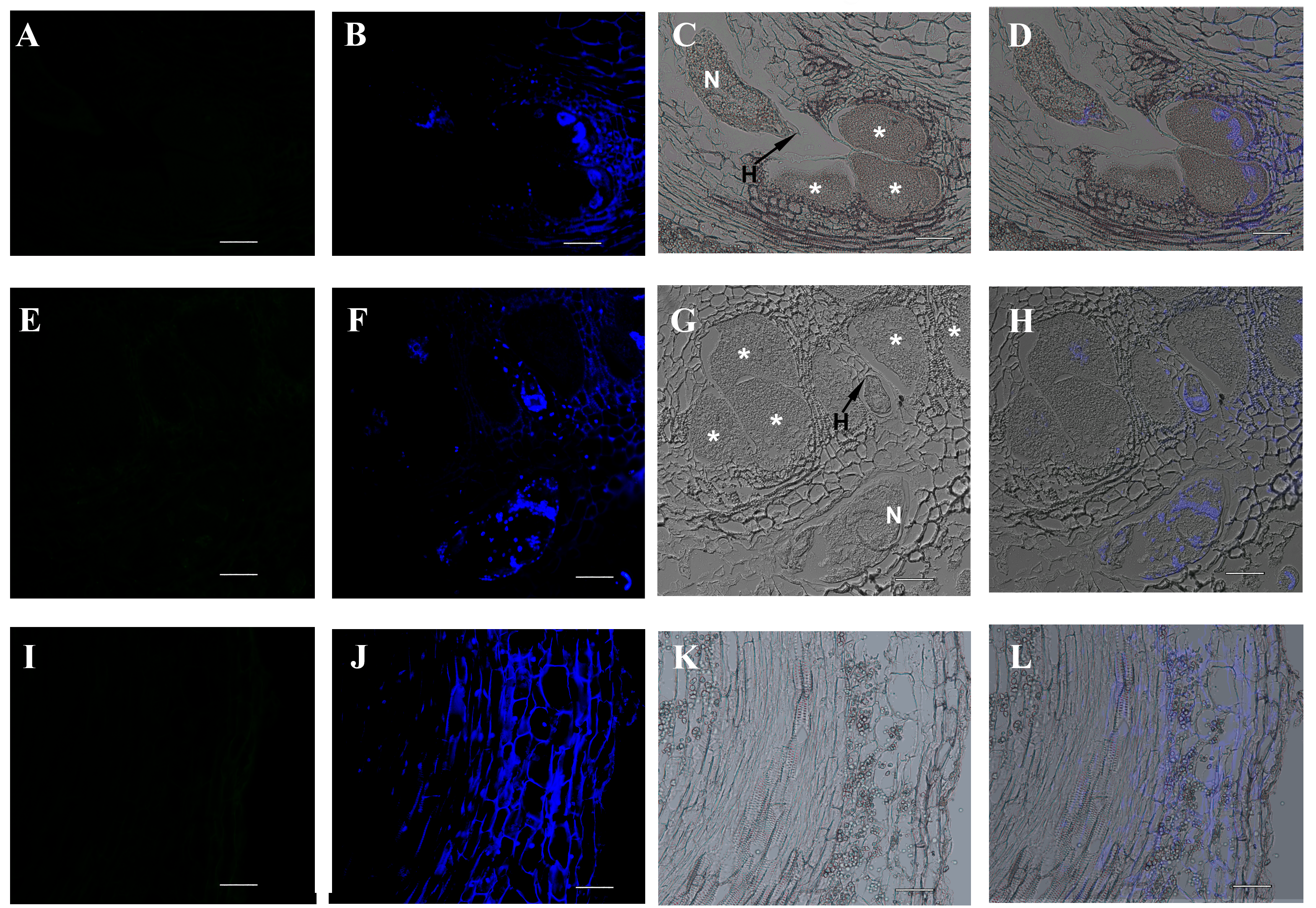

Supplement: S4 Fig — (A-D) Galls containing a nematode at 5 days postinfection (dpi) incubated with pre-immune serum, showing no signal. (E-H) Galls containing a nematode at 5 dpi without any treatment, showing no signal. (I-L) Healthy rice roots incubated with anti-MgGPP serum, showing no signal. Micrographs A, E and I are observations of the Alexa Fluor 488-conjugated secondary antibody. Micrographs B, F and J are images of 4,6-diamidino-2-phenylindole (DAPI)-stained nuclei. Micrographs C, G and K are images of differential interference contrast. Micrographs D, H and L are superpositions of images of the Alexa Fluor 488-conjugated secondary antibody, DAPI-stained nuclei and differential interference contrast. N, nematode; H, the head of nematode; asterisks, giant cells; Scale bars, 20 μm. (TIF) [file ppat.1006301.s005.tif]

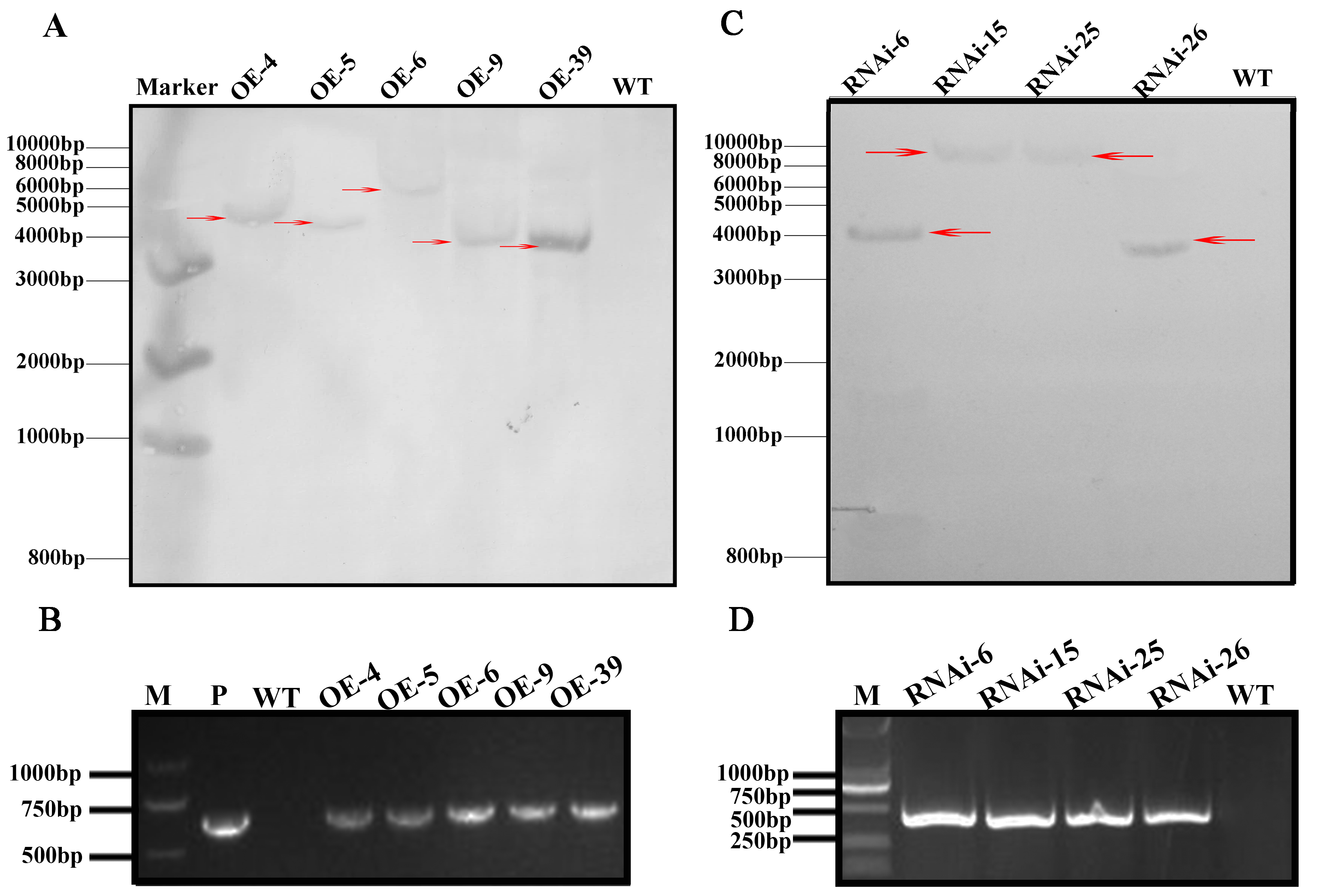

Supplement: S5 Fig — (A) and (C) Total gDNA was extracted from rice roots of overexpression and RNAi lines and wild-type (WT) controls. The genomic DNA was digested with the restriction endonuclease HindIII and then hybridized on blots with an MgGPP digoxigenin (DIC)-labeled probe, showing single-copy transgenic lines (red arrows). (B) and (D) RT-PCR was used to confirm the expression of MgGPP and the GUS intron in transgenic overexpression lines and RNAi lines compared with the WT control. OE-4, 5, 6, 9 and 39, five transgenic rice lines expressing MgGPP; P, positive control; WT, wild type. RNAi 6, 15, 25 and 26, different transgenic RNAi rice lines. M, standard molecular weight. (TIF) [file ppat.1006301.s006.tif]

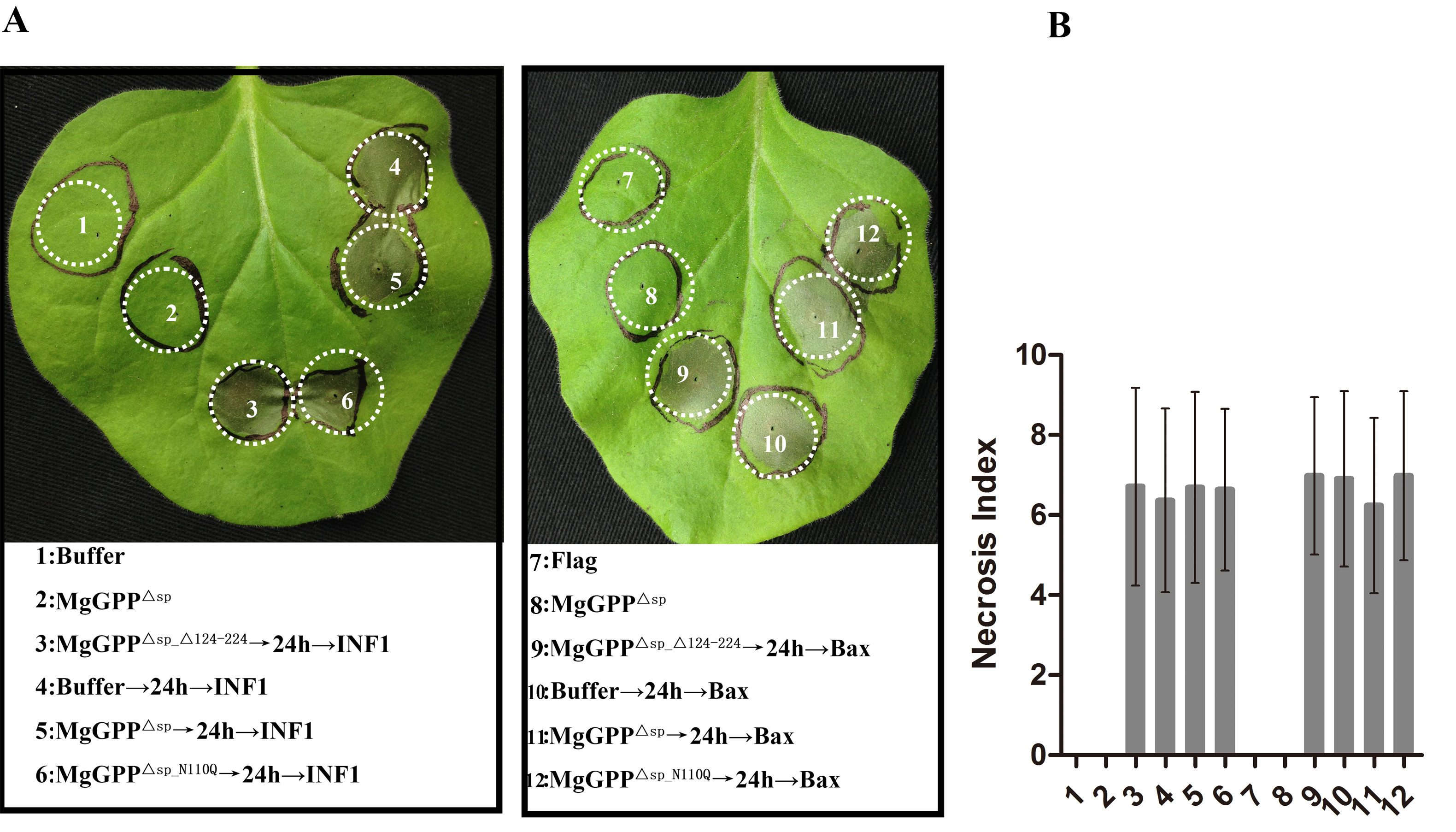

Supplement: S6 Fig — (A) Assay of the suppression of Bax- and INF1-triggered cell death in Nicotiana benthamiana by MgGPP. N. benthamiana leaves were infiltrated with buffer or Agrobacterium tumefaciens cells carrying MgGPPΔsp, MgGPPΔsp_Δ123–224, MgGPPΔsp_N110Q and the flag control gene alone or followed 24 h later with A. tumefaciens cells carrying the Bax or INF1 genes. The cell death phenotype was scored, and photographs were taken 5 days after the last infiltration. (B) The average areas of cell death of in leaves infiltrated with cells carrying MgGPP and other proteins followed by Bax or INF1. Statistical significance of the necrosis index of MgGPP and other proteins compared with that of the negative control flag. Each column represents the mean with standard deviation (n = 55). *P<0.05, **P<0.01, Student’s t test. (TIF) [file ppat.1006301.s007.tif]

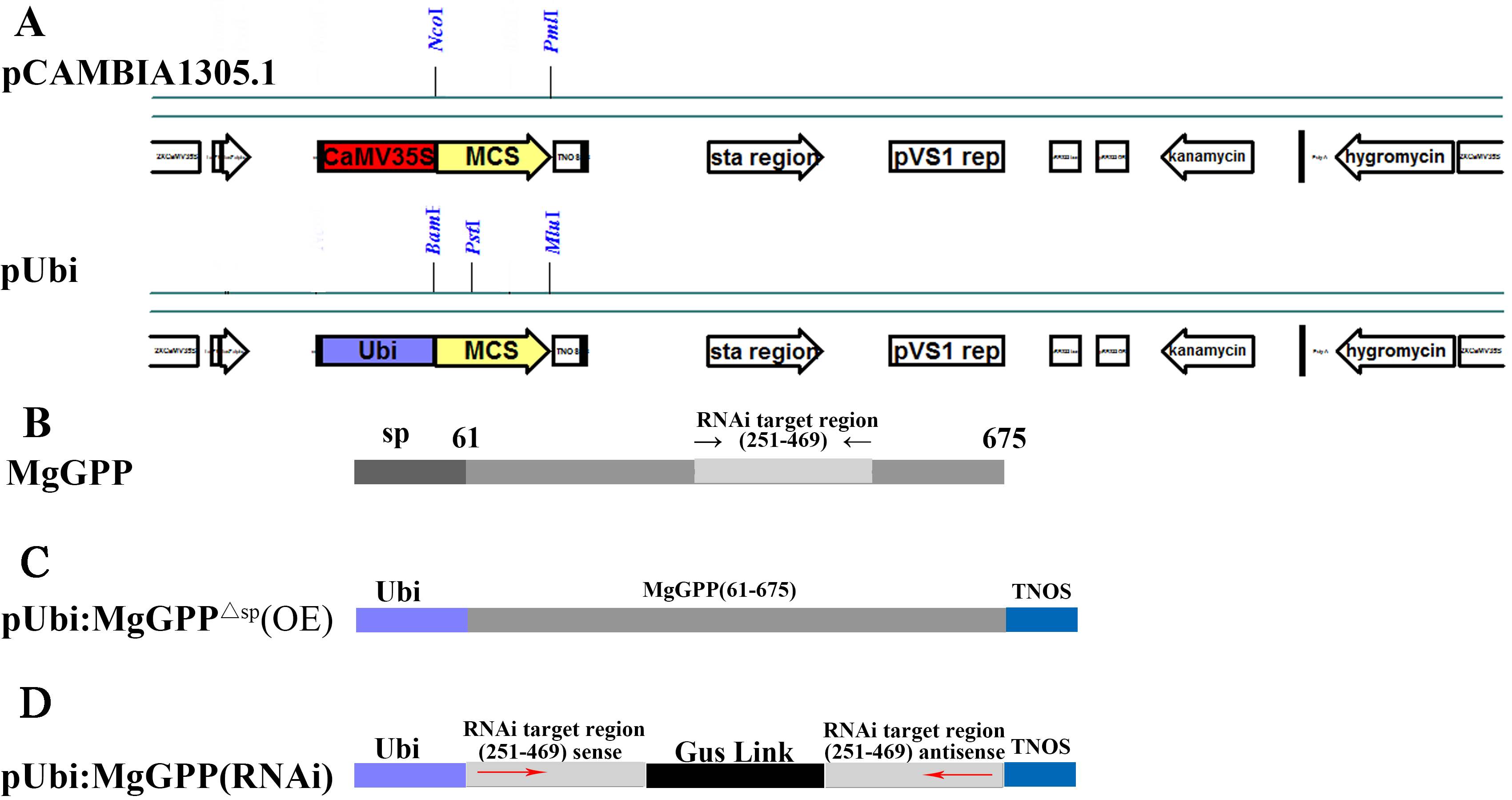

Supplement: S7 Fig — (A) The CaMV35S-promotor of pCAMBIA1305.1 vector was replaced with the maize ubiquitin promoter to generate the binary vector pUbi. (B) Schematic of the full-length MgGPP construct. (C) Constructs generated for MgGPP overexpression (OE) and (D) host-induced RNA interference (RNAi). (TIF) [file ppat.1006301.s008.tif]
